# FAM134B induces tumorigenesis and epithelial‐to‐mesenchymal transition via Akt signaling in hepatocellular carcinoma

**DOI:** 10.1002/1878-0261.12429

**Published:** 2019-01-24

**Authors:** Zhao‐qi Zhang, Jin Chen, Wan‐qiu Huang, Deng Ning, Qiu‐meng Liu, Chao Wang, Long Zhang, Li Ren, Liang Chu, Hui‐fang Liang, Hai‐ning Fan, Bi‐xiang Zhang, Xiao‐ping Chen

**Affiliations:** ^1^ Hepatic Surgery Center Tongji Hospital Tongji Medical College Huazhong University of Science and Technology Wuhan China; ^2^ Key Laboratory of Organ Transplantation Ministry of Education and Ministry of Public Health Wuhan China; ^3^ Clinical Medicine Research Center of Hepatic Surgery in Hubei Province Wuhan China; ^4^ Department of Biliary and Pancreatic Surgery Tongji Hospital Tongji Medical College Huazhong University of Science and Technology Wuhan China; ^5^ Department of Hepatopancreatobiliary Surgery Affiliated Hospital of Qinghai University Xining China

**Keywords:** Akt, epithelial‐to‐mesenchymal transition, FAM134B, HCC, RETREG1

## Abstract

*Fam134b* (JK‐1, RETREG1) was first identified as an oncogene in esophageal squamous cell carcinoma. However, the roles of FAM134B during tumorigenesis of hepatocellular carcinoma (HCC) and in epithelial‐to‐mesenchymal transition (EMT) were previously unclear. In this study, we investigated the function of FAM134B in HCC and the related tumorigenesis mechanisms, as well as how FAM134B induces EMT. We detected the expression of FAM134B in a normal hepatic cell line, HCC cell lines, fresh specimens, and a HCC tissue microarray. A retrospective study of 122 paired HCC tissue microarrays was used to analyze the correlation between FAM134B and clinical features. Gain‐ and loss‐of‐function experiments, rescue experiments, Akt pathway activator/inhibitors, nude mice xenograft models, and nude mice lung metastasis models were used to determine the underlying mechanisms of FAM134B in inducing tumorigenesis and EMT *in vitro* and *in vivo*. The expression level of FAM134B was highly elevated in HCC, as compared with that in normal liver tissues and normal hepatic cells. Overexpression of FAM134B was significantly associated with tumor size (*P* = 0.025), pathological vascular invasion (*P* = 0.026), differentiation grade (*P* = 0.023), cancer recurrence (*P* = 0.044), and portal vein tumor thrombus (*P* = 0.036) in HCC. Patients with high expression of FAM134B had shorter overall survival and disease‐free survival than patients with non‐high expression of FAM134B. Furthermore, knockdown of FAM134B with shRNAs inhibited cell growth and motility, as well as tumor formation and metastasis in nude mice, all of which were promoted by overexpression of FAM134B. Our study demonstrated that *Fam134b* is an oncogene that plays a crucial role in HCC via the Akt signaling pathway with subsequent glycogen synthase kinase‐3β phosphorylation, accumulation of β‐catenin, and stabilization of Snail, which promotes tumorigenesis, EMT, and tumor metastasis in HCC.

**Abbreviations**
CCK8: cell counting kit‐8
EMT: epithelial‐to‐mesenchymal transition
ER: endoplasmic reticulum
H&E: hematoxylin and eosin
HCC: hepatocellular carcinoma
IF: immunofluorescence
IHC: immunohistochemistry

## 1 Introduction

According to the International Agency for Research on Cancer, hepatocellular carcinoma (HCC) is the fifth most common cancer in men and the seventh in women worldwide (Forner *et al*., 2018). Even with the advancement of early diagnosis and treatments, the 5‐year survival for HCC patients has not improved much over the last few decades (Coleman, 2014). Uncontrolled cell proliferation and metastasis are the main obstacles to clinical management of HCC (Allemani *et al*., 2015; Reeves and Aisen, 2015). Therefore, discovery of the mechanisms underlying tumor progression is crucial to develop new strategies for the treatment of HCC.

Epithelial‐to‐mesenchymal transition (EMT) plays an important role in embryonic development as well as the motility and metastasis of tumor cells (De and Berx, 2013). EMT interrupts intercellular adhesion and reorganization of the actin cytoskeletal, which enhances the migration and invasion abilities of cancer cells (Chausovsky *et al*., 2000; Gumbiner, 2005). E‐cadherin expressed abundantly in most epithelial cells, but it is decreased at cell junctions during EMT (Jawhari *et al*., 1997), which is related to the metastasis of epithelial tumor cells by mediating of intercellular adhesion and acquiring mesenchymal phenotype, then facilitating cellular migration and invasion (Seidel *et al*., 2004). Snail induces EMT by inhibiting the transcription of E‐cadherin (Batlle *et al*., 2000; Cano *et al*., 2000).

Akt is a serine and threonine kinase that plays a key role in cell‐to‐cell signaling pathway during tumorigenesis (Vivanco and Sawyers, 2002). Akt, regulated by many upstream factors, has numerous downstream targets with various functions, such as glycogen synthase kinase‐3 (GSK‐3), FoxO, and mTORC1, which extremely expand the functionality of Akt. This complex and dynamic network of the Akt signaling pathway, allows for the distribution of Akt to almost every cell in the body (Manning and Toker, 2017). GSK‐3 plays an essential part in maintaining the epithelial phenotype, while persistent phosphorylation of GSK‐3β is critical for the growth regulation of hepatoma cells (Desbois‐Mouthon *et al*., 2002). GSK‐3β inhibits the expression of Snail endogenously by inhibiting expression of the transcription factor nuclear factor κB (Bachelder *et al*., 2005) and degrades constitutive β‐catenin in the cytoplasm (Mosimann *et al*., 2009). The activation of Akt phosphorylates GSK‐3β (Fukumoto *et al*., 2001), thereby reducing the transcriptional repression of Snail (Zhou *et al*., 2004) and decreasing the degradation of β‐catenin, which leads to the accumulation of both Snail and β‐catenin (Mao *et al*., 2009; Sharma *et al*., 2002; Zhou *et al*., 2004). β‐catenin activates transcription from the cyclin D1 promoter, which produces high levels of cyclin D1 messenger RNA and protein constitutively (Tetsu and Mccormick, 1999), and results in a shift in cell cycle from the G1 phase to S phase (Liang and Slingerland, 2003).

The *Fam134b* gene, located on chromosome 5p15.1, was first identified as a regulator of the malignant phenotype and a downstream molecule of δ‐catenin in esophageal squamous cell carcinoma (Tang *et al*., 2007). However, in colon cancer, FAM134B inhibits the migration of tumor cells (Kasem *et al*., 2014a,b). The aims of this study were to investigate the tumorigenic and metastatic roles of FAM134B in the development of HCC both *in vivo* and *in vitro*, and to identify the underlying mechanisms of FAM134B on cell proliferation and metastasis in HCC.

## 2 Materials and methods

### 2.1 Patients and HCC tissue specimens

A total of 50 paired specimens of tumor and adjacent non‐tumor tissues were collected from 50 HCC patients who underwent hepatectomy at the Hepatic Surgery Center, Tongji Hospital of Huazhong University of Science and Technology (HUST; Wuhan, China). Matched fresh specimens of HCC tissues and adjacent non‐tumorous liver tissue were lysed separately for western blot analysis. A tissue microarray of 122 pairs of primary HCC tissues with their clinical and prognosis data were acquired from the specimen library of Hepatic Surgery Center, Tongji Hospital of HUST. The experiments were undertaken with the understanding and written consent of each subject. The study methodologies conformed to the standards set by the Declaration of Helsinki. All procedures performed in this study involving human participants were approved by the Ethics Committee of Tongji Hospital.

### 2.2 Cell lines and culture

All cell lines (HL‐7702, Bel‐7402, Huh7, PLC8024, HepG2, Hep3B, HLF, SMMC7721 and 293T) were purchased from the China Center for Type Culture Collection (Wuhan, China) and cultured in Dulbecco's modified Eagle's medium (Invitrogen Corporation, Carlsbad, CA, USA) supplemented with 10% FBS (Life Technologies Inc., Gibco/Brl Division, Grand Island, NY, USA) in a humidified atmosphere of 5% CO_2_ at 37 °C. According to the ATCC database, the morphology of PLC8024, HepG2 and Hep3B is epithelial. SMMC7721, Bel7402, and Huh7 cell lines have low invasiveness and metastatic proclivity (Zeng *et al*., 2017), while HLF cell line has highly aggressive behavior (Leng *et al*., 2016). Cell lines were authenticated by array comparative genomic hybridization or short tandem repeat DNA profiling were performed within < 10 passages after authentication and < 20 passages after receipt from commercial suppliers.

### 2.3 Chemicals, inhibitors and antibodies

Puromycin, G418, LY294002, FR180204 and SC79 were purchased from Cayman Chemical Company (Ann Arbor, MI, USA). The following antibodies were used for either western blot or immunohistochemical analysis: FAM134B (ab151755; Epitomics, Burlingame, CA, USA), JNK (ab110724; Epitomics), FAM134B (#ARP44827; Aviva Systems Biology, San Diego, CA, USA), E‐cadherin (610181; BD Biosciences, San Jose, CA, USA), and GAPDH (KC‐5G4; KangChen Bio‐tech, Inc., Shanghai, China). The following antibodies were purchased from Cell Signaling Technology, Inc. (Beverly, MA, USA): Akt (C67E7), phospho‐Akt (Thr308; D25E6), phospho‐Akt (Ser473; D9E), Erk (137F5), phospho‐Erk (D13.14.4E), p38 (D13E1), phospho‐p38 (3D7), phospho‐JNK (Thr183/Tyr185), p70s6k (49D7), phospho‐p70s6k (1A5), Snail (C15D3), cyclin D1 (92G2), GSK‐3β (D75D3), phospho‐GSK‐3β (Ser9; D3A4), and β‐catenin (D10A8), Occludin (D15G7), Vimentin (D21H3), N‐Cadherin (D4R1H).

### 2.4 Western blot analysis

Western blot analysis was performed as described previously (An *et al*., 2016) and the results were quantified using the image analysis tool imagej (National Institutes of Health, Bethesda, MD, USA). Each experiment were repeated three times.

### 2.5 Real‐time PCR

Real‐time PCR was performed as described previously (Chen *et al*., 2014). The following primers used for quantitative reverse transcription–PCR of Snail: 5′‐TCG GAA GCC TAA CTA CAG CGA‐3′ (forward) and 5′‐AGA TGA GCA TTG GCA GCG AG‐3′ (reverse). The Ct values of Snail were equilibrated to those of the internal control GAPDH. Relative expression was calculated using the 2^−ΔΔCt^ method. Each experiment were repeated three times.

### 2.6 Plasmids, lentivirus and stable cell lines

Full‐length human FAM134B cDNA was amplified by PCR and subcloned into the lentiviral vector pBABE‐puro (plasmid # 1764; Addgene, Cambridge, MA, USA) to establish a Bel‐7402 cell line that stably overexpressed FAM134B. The target sequences of FAM134B were used to establish a stable HLF cell line with FAM134B knockdown. Briefly, three DNA fragments (FAM134B shRNA#1 CCG GGC AGC TAT CAA AGA CCA GTT A, FAM134B shRNA#2 CCG GCC ACA GAC AGA CAC TTC TGA T, and FAM134B shRNA#3 CCG GCT ACT GTT ACT GTG TGC ATT T) were subcloned into the lentiviral vector pLKO.1 neo (plasmid # 13425; Addgene). The target sequences of Snail shRNA#1 and #2 were AAC TGC AAA TAC TGC AAC A and ACT CAG ATG TCA AGA AGT A, respectively. The target sequences of β‐catenin siRNA#1 and #2 were AGC UGA UAU UGA UGG ACA G and CAG UUG UGG UUA AGC UCU U, respectively. The plasmids pMD2.G and psPAX2 were gifts from Didier Trono (plasmids # 12259 and # 12260; Addgene). To obtain stable cell clones, HCC cells were infected with lentivirus for 24 h and selected with growth medium containing 5 μg·mL^−1^ of puromycin for 3 days and/or 400 μg·mL^−1^ of G418 for 7 days. The stability of the transfected clones was validated by western blot analysis.

### 2.7 Cell viability assays

For the cell counting kit‐8 (CCK8) assay (Sigma‐Aldrich Corporation, St. Louis, MO, USA), indicated cells were seeded in 96‐well plates for 5–6 days with replacement of the culture media every 2 days. CCK8 solution was added to the wells of the plates for 1–2 h to test the optical density value at 450 nm (Elx 800; BioTek Instruments, Inc., Winooski, VT, USA). For the colony formation assay, cells (500 cells per well) were plated in six‐well plates with replacement of the culture media every 2 days. After 2 weeks, the plates were stained with 1% crystal violet (Sigma‐Aldrich Corporation) and photographed. Colonies were counted and analyzed using the Alpha Innotech Imaging system (Alphatron Asia Pte, Ltd, Singapore, Singapore). Each experiment were repeated three times.

### 2.8 Cell cycle and apoptosis assay

Flow cytometry was performed as described previously (Wei *et al*., 2012). For the cell‐cycle assay, the indicated cells were modified and harvested as follows. A total of 1 × 10^6^ cells per sample were analyzed for cell‐cycle distribution using an FACS Aria Cell Cytometer (BD Biosciences). For the apoptosis assay, 2 × 10^5^ cells per sample were harvested and tested. All data were analyzed using Cell Quest software (BD Biosciences). Each experiment were repeated three times.

### 2.9 Wound healing and transwell assays

For the wound healing assay, the indicated cells were cultured on a 35‐mm dish until confluence and then wounded with a 10‐μL pipette tip. Migration photos were captured at 0, 24, and 48 h after scratching. The transwell migration and invasion assay was performed as described previously (Ding *et al*., 2014). Each experiment were repeated three times.

### 2.10 Immunohistochemistry (IHC) and immunofluorescence (IF) analyses

Immunohistochemistry (IHC) analysis was performed as previously described (Yoo *et al*., 2010). The microscopic examination of each point of the tissue microarray is performed at the same incident light intensity and compensation intensity. The total score for each point is the product of the staining intensity score and the stained positive cells score. The rule of staining intensity score: 0 point (Negative); 1 point (Light brown); 2 points (Brown); 3 point (Dark brown). The rule of stained positive cells score: 0 point (0%); 1 point (10–25%); 2 points (26–50%); 3 point (51–75%); 4 point (76–100%). We define it as positive if the total score is ≥ 6 points, otherwise we define it as negative. The total score of the tissue chip was independently completed by two pathologists who had no knowledge of the patient's clinical case data. Immunofluorescence (IF) staining was performed according to a previously reported method (An *et al*., 2016). FAM134B antibody was diluted to 1 : 100. Nuclei were labeled with 4′,6‐diamidino‐2‐phenylindole. Each experiment were repeated three times.

### 2.11 Xenograft tumorigenicity assay

Four‐week‐old male BALB/c (nu/nu) mice were bred under specific pathogen‐free conditions. All animals were cared according to the Guide for the Care and Use of Laboratory Animals at the Laboratory Animal center of the Tongji Medical College, HUST. The experimental protocol was approved by the Committee on the Ethics of Animal Experiments of the Tongji Medical College, HUST. The mice were euthanized if they showed any adverse signs or symptoms of diseases which were beyond our intention in the experimental protocol. For the tumorigenicity assay, 1 × 10^6^ tumor cells suspended in phosphate‐buffered saline were injected subcutaneously into the flanks of the nude mice (*n* = 10 per group). The length and width of the tumors were measured with a vernier caliper every 3 days after injection, and the tumor volume was calculated according to the formula *V* = *L* × *W* ^2^ × 0.5.

### 2.12 Lung metastases assay *in vivo*

The lung metastases assay was performed as previously reported (Lin *et al*., 2017). Briefly, six mice in the experiment group were injected intravenously with 1 × 10^6^ tumor cells suspended in phosphate‐buffered saline via the lateral tail vein, while six mice in the control group were injected intravenously with the same volume phosphate‐buffered saline via the lateral tail vein. All mice were sacrificed after 8 weeks and the lung tissues were removed and fixed in 4% phosphate‐buffered neutral formalin for 72 h. Then the metastatic lungs were longitudinal sectioned every 0.5 mm, ~ 20 slices can be cut for each lung. After hematoxylin and eosin (H&E) staining, metastases was quantified by counting the metastatic nodules using blind method.

### 2.13 Statistical analysis

All data are presented as the mean ± standard error of mean (SEM). Statistical analysis was conducted using prism 6.0 software (GraphPad Software, Inc., La Jolla, CA, USA). Categorical data were analyzed using the chi‐square test or Fisher's exact test. Quantitative data were compared using the two‐tailed Student's *t*‐test, one‐factor analysis of variance or two‐factor analysis of variance (One‐way ANOVA or Two‐way ANOVA) with the Wilcoxon signed‐rank test. A two‐tailed probability (*P*) value of < 0.05 was considered statistically significant.

## 3 Results

### 3.1 Frequent upregulation of FAM134B in HCC

**Figure 1:**
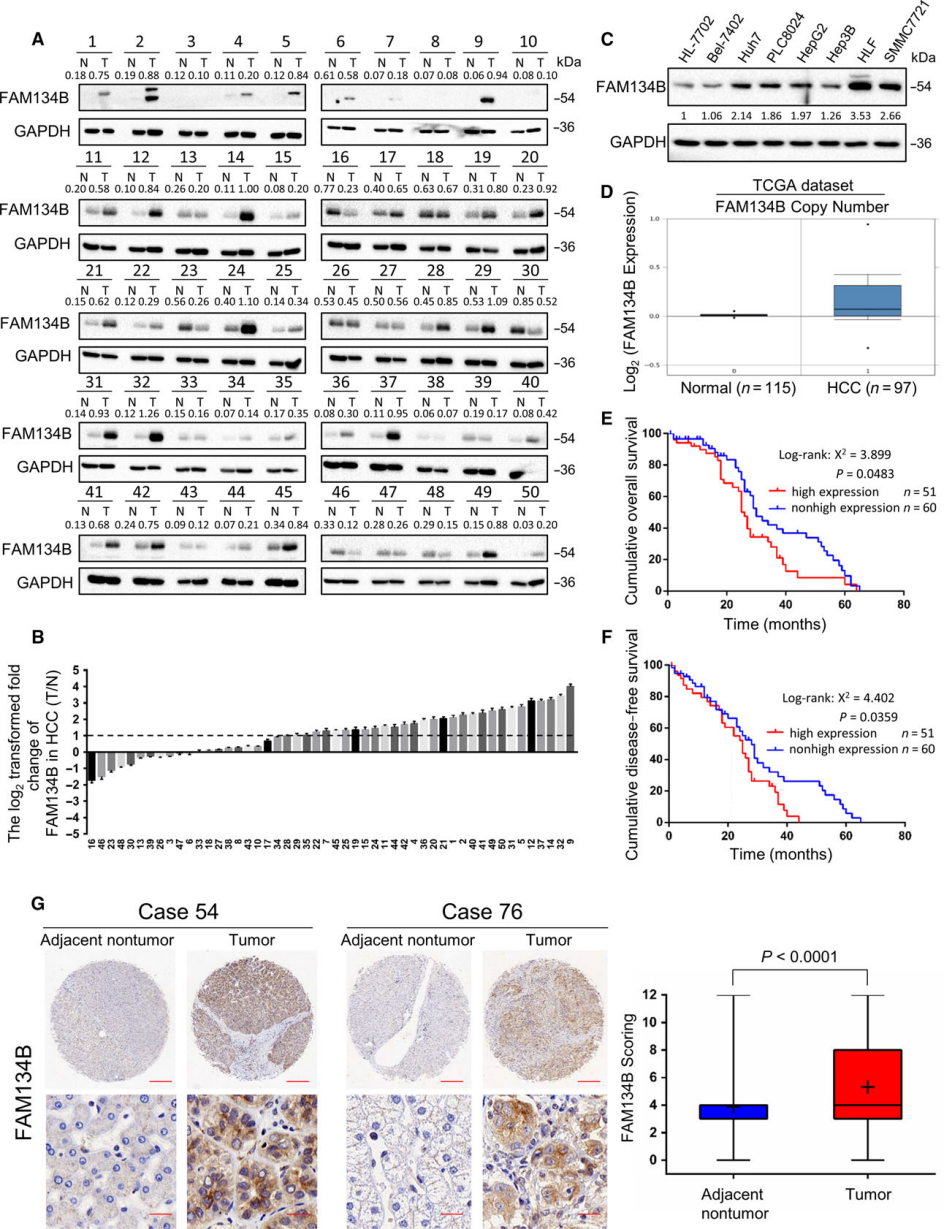
FAM134B is overexpressed in HCC. (A) Western blot analysis of FAM134B protein levels in 50 pairs of HCC tissues and adjacent non‐tumor tissues selected randomly. GAPDH was used as a loading control. (B) Western blot analysis of relative FAM134B expression in 50 HCC tissues (T) and adjacent non‐tumor tissues (N). *Y* axis represents the log2 transformed fold change in the T/N protein expression ratio of FAM134B. The number of each specimen is indicated below the *x* axis. (C) Western blot analysis of FAM134B expression in one normal hepatic cell line and seven HCC cell lines. GAPDH was used as a loading control. (D) Comparison of FAM134B DNA copy number in normal and HCC tissues. A box plot was derived from gene expression data retrieved from The Cancer Genome Atlas dataset in ONCOMINE. Kaplan–Meier's analysis of correlations between OS (E) or diseases‐free survival (F) of 111 HCC patients (11 patients are lost to follow‐up) and FAM134B expression level. Based on IHC staining analysis of the tissue microarray, HCC patients were divided into FAM134B high expression (*n* = 51) and FAM134B non‐high expression (*n* = 60) groups. (*P* < 0.05, log‐rank test). (G) IHC analysis of FAM134B expression in 122 paired HCC tissues. Representative images of FAM134B expression in a matched primary HCC sample and its adjacent non‐tumor tissue are in the left panels. FAM134B scoring analysis is in the right panel by independent Student's *t*‐test. Scale bar, 300 μm (upper panel) or 20 μm (lower panel).

FAM134B protein expression in 50 pairs of HCC tumor versus adjacent non‐tumor tissues was evaluated by western blot analysis (Fig. 1A). The log2 transformed fold change of FAM134B in HCC showed that overexpression of FAM134B (defined as > 2‐fold increase) was detected in 31/50 (62%) pairs of primary HCC tumor versus adjacent non‐tumor tissues (Fig. 1B). Western blot analysis showed that FAM134B expression was low in the HL‐7702, Hep3B and Bel‐7402 cell lines, and overexpressed in other five cell lines (Fig. 1C). In the ONCOMINE cancer microarray database (https://www.oncomine.org/resource/login.html), the copy numbers of FAM134B were greater in the HCC group than the normal group (Fig. 1D). A tissue microarray of 122 pairs of primary HCC tissues was tested by IHC analysis. Overexpression of FAM134B was detected in 56/122 (45.9%) of HCC tumor tissues as compared with the adjacent non‐tumor tissues, and there were 45.1% (55/122) HCC tumor tissues showed no significant difference in the expression of FAM134B, but there were only 9% (11/122) HCC tumor tissues were low expressed FAM134B. (Fig. 1G).

### 3.2 Clinical significance of FAM134B overexpression in HCC

To investigate the clinical significance of FAM134B expression, we first measured its expression in a tissue microarray of 122 paired HCC and adjacent non‐tumor tissue samples with clinicopathological features (Table 1). IHC assays showed that the average expression level of FAM134B was significantly higher in HCCs than that in adjacent non‐tumor tissues (Fig. 1G). Up‐regulation of FAM134B was confirmed in an additional 50 paired HCC samples using western blotting (Fig. 1A). Patients were divided according to the expression of FAM134B (high expression or non‐high expression).We then performed western blotting to measure expression of FAM134B in liver cell lines and human HCC cell lines with varying cell context and metastatic capability. We found that overexpression of FAM134B was observed in most of HCC cell lines with mesenchymal cell lines (HLF and SMMC7721), compared to that in normal liver cell lines (HL‐7702; Fig. 1C) as well as epithelial cell lines (Huh7, PLC8024, HepG2 and Hep3B). This finding was further validated by clinicopathological analysis of FAM134B expression in 122 paired HCC samples. Chi squared analysis indicated that overexpression of FAM134B was significantly associated with tumor size (*P* = 0.025), pathological vascular invasion (*P* = 0.026), differentiation grade (*P* = 0.023), cancer recurrence (*P* = 0.044) and portal vein tumor thrombus (*P* = 0.036) in HCC (Table 1). Kaplan–Meier's analysis showed that patients with high expression of FAM134B had shorter overall survival (OS) and shorter disease‐free survival than patients with non‐high expression of FAM134B (Fig. 1E,F). Taken together, these data indicated that the expression of FAM134B is upregulated in HCC. High expression of FAM134B predicts poor prognosis in HCC patients and may contribute to progression of HCC.

**Table 1:** Clinicopathological correlation of FAM134B expression in HCC

Characteristics	Total	Non‐high expression of FAM134B	High expression of FAM134B	*P*
Gender				
Male	103	58	45	0.254
Female	19	8	11	
Age				
≤ 55 years	77	40	37	0.533
> 55 years	45	26	19	
Serum alpha‐fetoprotein				
< 400 ng·mL^−1^	65	36	29	0.761
≥ 400 ng·mL^−1^	57	30	27	
Tumor size^a^				
< 5 cm	48	32	16	**0.025**
≥ 5 cm	74	34	40	
Cirrhosis				
Absent	33	14	19	0.115
Present	89	52	37	
Tumor encapsulation				
Absent	56	32	24	0.534
Present	66	34	32	
Adjacent organ invasion				
Absent	85	45	40	0.697
Present	37	21	16	
Pathological vascular invasion				
Absent	74	46	28	**0.026**
Present	48	20	28	
Cancer recurrence				
Absent	60	38	22	**0.044**
Present	62	28	34	
Differentiation grade				
High grade	29	21	8	**0.023**
Low grade	93	45	48	
Portal vein tumor thrombus				
Absent	101	59	42	**0.036**
Present	21	7	14	

a Tumor size was measured by the length of the largest tumor nodule. *P* values of the characteristics with statistical significant were bolded.

### 3.3 FAM134B promotes cell proliferation and tumorigenesis in HCC

**Figure 2:**
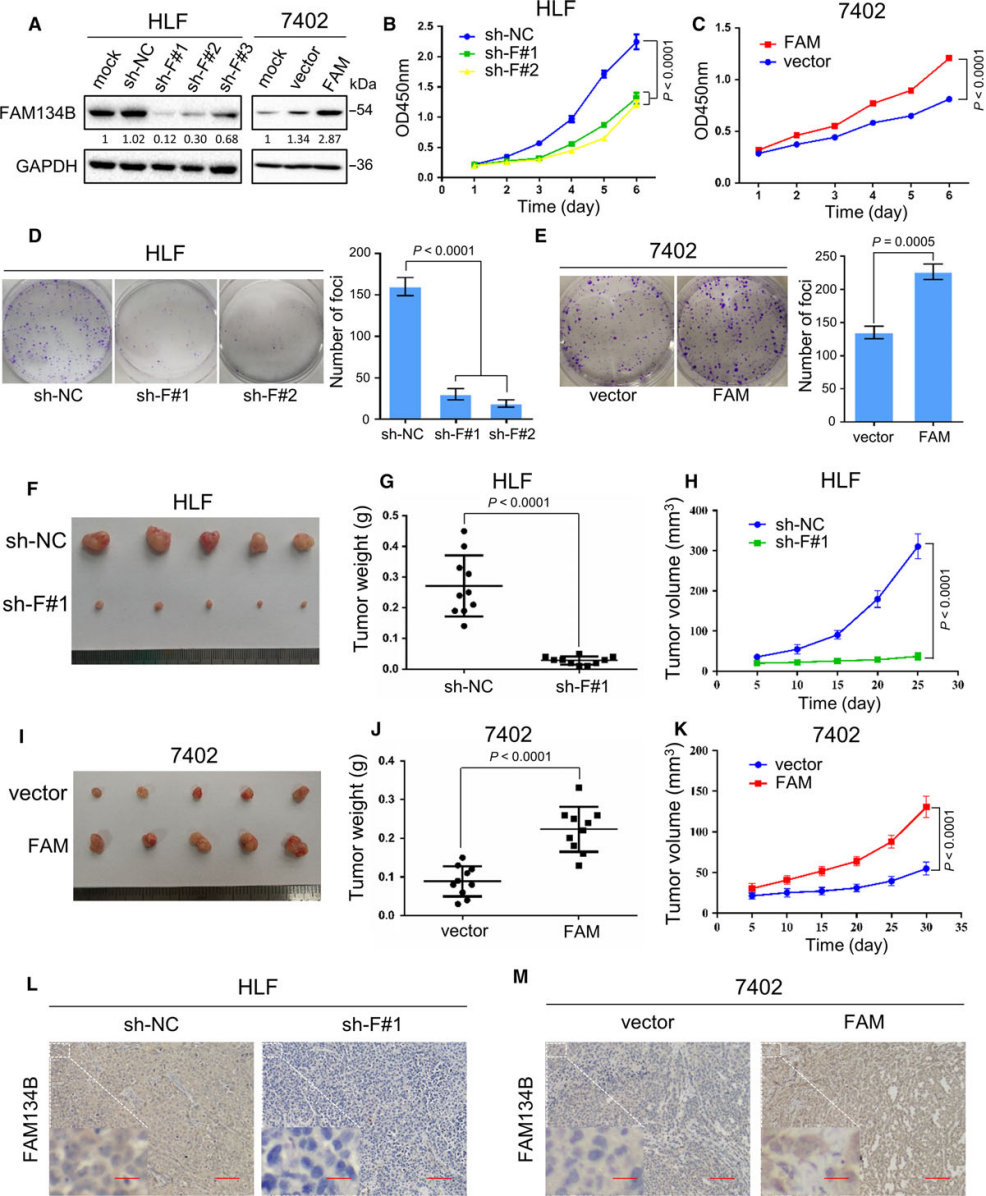
FAM134B promotes proliferation and tumorigenesis of HCC cells *in vitro* and *in vivo*. (A) Three shRNAs (sh‐F#1‐3) against FAM134B effectively decreased FAM134B expression in HLF cells, while FAM134B was stably overexpressed in Bel‐7402 cells as detected by western blot analysis. Transfection with scrambled shRNA (sh‐NC) and empty vectors (vector) were used as negative controls, and GAPDH was used as a loading control. Knockdown of FAM134B expression effectively inhibited cell growth (B), foci formation (D), and tumor formation in nude mice (F). Overexpression of FAM134B promoted cell growth (C), foci formation (E), and tumor formation in nude mice (G). The results are expressed as the mean ± SEM of three independent experiments. Tumor weight (H, I) and tumor volume (J, K) are shown as means ± SEM. (L, M) Representative IHC images of FAM134B expression in xenograft tumors. Scale bar, 100 μm (right) or 20 μm (magnified image on the left). Independent Student's *t*‐test was used in panel (E, G, and J). One‐way ANOVA was used in panel (D). Two‐way ANOVA was used in panel (B, C, H, and K).

To determine whether FAM134B promotes tumorigenesis, HLF cells were stably transfected with three shRNAs against FAM134B and named HLF sh‐FAM134B#1 (abbreviate as sh‐F#1), HLF sh‐FAM134B#2 (sh‐F#2), and HLF sh‐FAM134B#3 (sh‐F#3), respectively, with the use of scrambled shRNA‐transfected cells (sh‐NC) as negative controls. Bel‐7402 (7402) cells were stably transfected with the FAM134B construct (7402 FAM) with empty vector‐transfected (abbreviate as vector) used as negative controls. The effects of overexpression and knockdown was detected by western blot analysis. As shown in Fig. 2A, HLF sh‐F#1 and sh‐F#2 showed significant knockdown effects, so these two cell lines were chosen to perform the following experiments. A cell line overexpressing FAM134B was successfully constructed. Functional assays were used to characterize the tumorigenicity of FAM134B. The results of the CCK‐8 assay showed that the growth rate of FAM134B‐knockdown cells was less than that of the control cells (*P *<* *0.01, Fig. 2B), while the growth rate of FAM134B‐transfected cells was greater than that of the control cells (*P *<* *0.01, Fig. 2C). The foci formation assay showed a lower number and smaller colonies (*P *<* *0.001) in the FAM134B‐knockdown cells and a higher number and larger colonies (*P *<* *0.01) in the FAM134B‐transfected cells, as compared to the control cells (Fig. 2D,E, respectively).

Subcutaneous tumor formation in 10 nude mice was induced by subcutaneous injection of HLF FAM134B‐knockdown and FAM134B‐transfected 7402 cells into the left dorsal flank. The same number of HLF sh‐NC cells and 7402 vector cells were injected into the left dorsal flank of each mouse as a control. After 25 and 30 days, both groups of mice were sacrificed. The size, weight, and volume of the xenograft tumors were measured. The results showed that tumors developed from FAM134B‐knockdown cells were significantly smaller (Fig. 2F,J, *P* < 0.0001) and lighter (Fig. 2H, *P* < 0.0001), while those developed from FAM134B‐transfected cells were significantly larger (Fig. 2G,K, *P* < 0.001) and heavier (Fig. 2I, *P* < 0.001) than tumors from control cells. The expression of FAM134B from xenograft tumors that developed from the FAM134B‐knockdown cells and FAM134B‐transfected cells was confirmed by IHC staining (Fig. 2L,M) and the H&E stain (Fig. S1).

### 3.4 FAM134B activates the Akt/GSK‐3β/β‐catenin pathway

The strong ability of FAM134B to induce tumorigenesis was investigated. In the classic signaling pathways, knockdown of FAM134B inhibited the Akt signaling pathway and activated Erk signaling pathway, while the p38, p70s6k and JNK signaling pathways were not affected (Fig. S2). In order to identify which signaling pathway plays a critical role in the process of tumorigenesis induced by FAM134B, the Erk inhibitor FR180204 was used to investigate whether the upregulation of p‐Erk induced by FAM134B‐knockdown was involved in the decrease in cell proliferation. The results showed that activation of the Erk pathway had no significant effect on the decrease in cell proliferation caused by FAM134B knockdown (Fig. S3).

**Figure 3:**
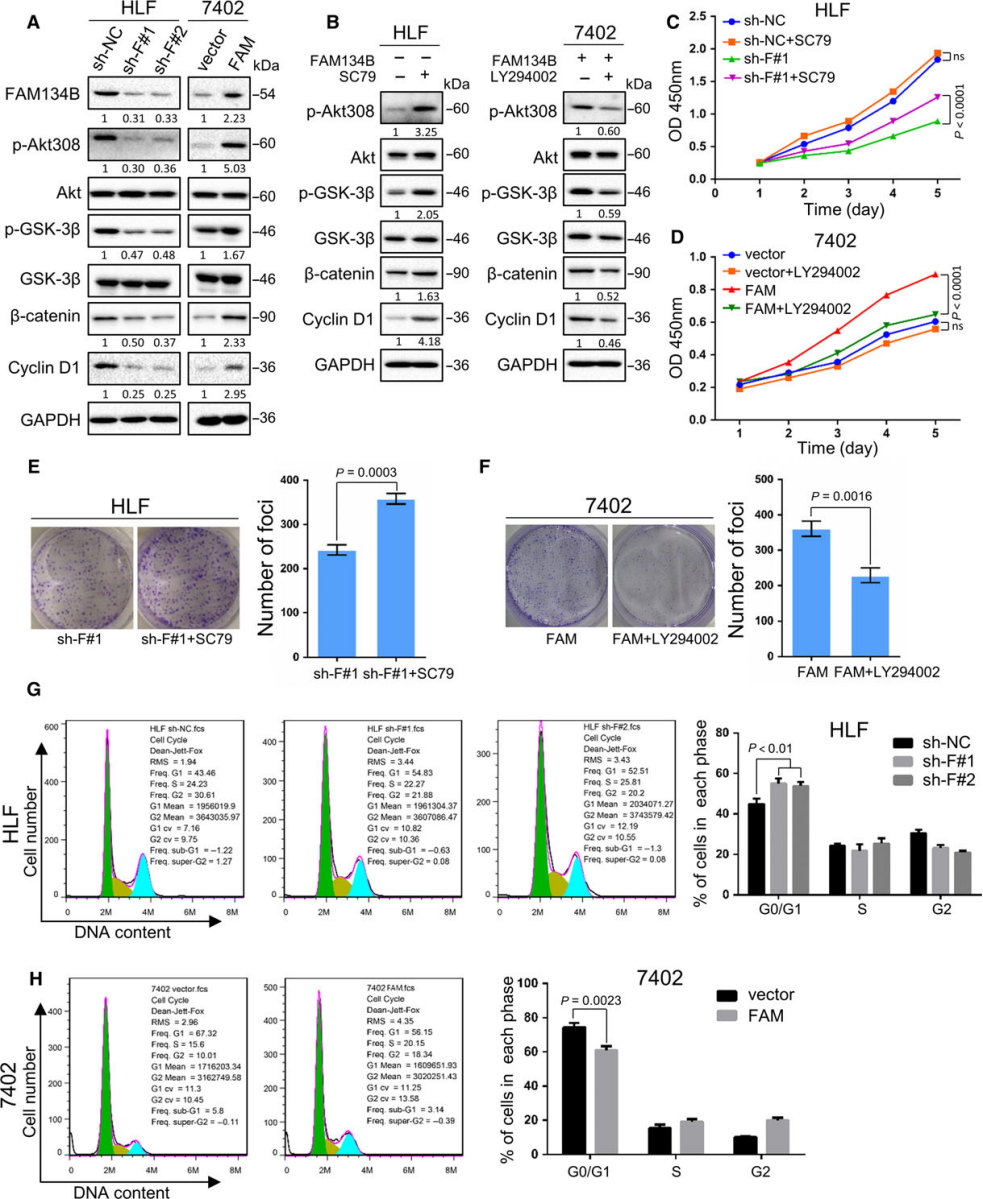
FAM134B activation of the Akt/GSK‐3β/β‐catenin pathway promoted cell proliferation. Western blot analysis comparing FAM134B‐transfected and FAM134B‐knockdown cells (A) as well as FAM134B‐knockdown cells treated by SC79 (10 μm) and FAM134B‐transfected cells treated with LY294002 (10 μm). (B) The relative expression levels of phosphorylated Akt308, Akt, phosphorylated GSK‐3β, GSK‐3β, β‐catenin, and cyclin D1 with respective control cells. GAPDH was used as a loading control. SC79 promoted cell growth (C) and foci formation (E) in FAM134B‐knockdown cells, while LY294002 inhibited cell growth (D) and foci formation (F) in FAM134B‐transfected cells. The results are expressed as the mean ± SEM of three independent experiments. Cell cycle arrest after overexpression (G) and knockdown (H) of FAM134B in Bel‐7402 and HLF cells, respectively, was assessed by flow cytometry. Two‐way ANOVA was used in panel (C and D), Independent Student's *t*‐test was used in panel (E, F, and H), One‐way ANOVA was used in panel (G).

FAM134B‐knockdown inactivated phosphorylated Akt, as well as its downstream phosphorylated GSK‐3β, β‐catenin and cyclin D1 in FAM134B‐knockdown HLF cells, while an opposite expression pattern of these proteins was observed in FAM134B‐transfected 7402 cells (Fig. 3A).

To confirm that the oncogenic effect of FAM134B was induced by activation of the Akt/GSK‐3β/β‐catenin axis, the Akt activator SC79 and the PI3K inhibitor LY294002 were used to explore the correlation between Akt and cell proliferation induced by FAM134B. The results showed that SC79 effectively increased the expression levels of phosphorylated Akt, phosphorylated GSK‐3β, and β‐catenin induced by FAM134B‐knockdown in HLF cells (Fig. 3B). An opposite expression pattern of these proteins was observed after LY294002 was used in FAM134B‐transfected 7402 cells (Fig. 3B). SC79 could reverse the ability of cell growth (Fig. 3C) and foci formation (Fig. 3E), which were decreased after FAM134B knockdown, while LY294002 can also reverse the ability of cell growth (Fig. 3D) and foci formation (Fig. 3F) acquired by transfection with FAM134B.

As FAM134B affected cell growth and foci formation, we wanted to determine whether FAM134B was involved in cell apoptosis or cell cycle. The results showed that FAM134B‐knockdown did not influence cell apoptosis (Fig. S4) but did influence cell cycle. HLF cells were arrested in the G1 phase after FAM134B knockdown (Fig. 3G) and the number of 7402 cells in the G1 phase was decreased by FAM134B over‐expression (Fig. 3H), which is correlated with the expression of cyclin D1 (Takahashiyanaga and Sasaguri, 2008).

### 3.5 FAM134B promotes cell proliferation by accumulation of β‐catenin and activation of cyclin D1

**Figure 4:**
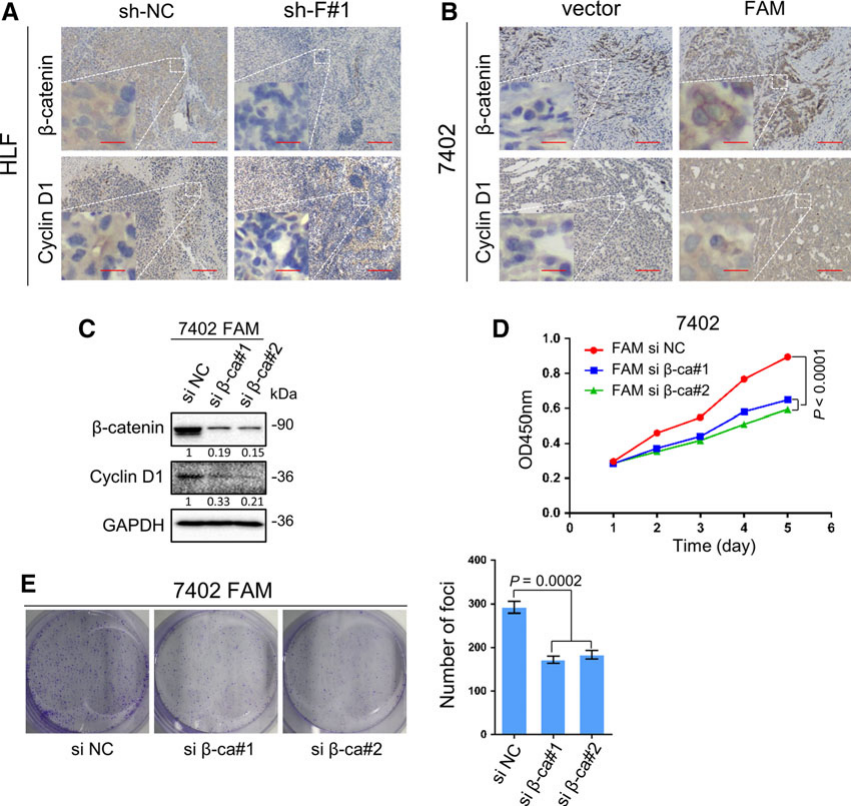
FAM134B promotes cell proliferation by accumulation of β‐catenin and activation of cyclin D1. Representative IHC staining of β‐catenin and cyclin D1 expression in HLF FAM134B knockdown cells (A) and 7402 FAM134B overexpressing cells (B) with respective control cells. Scale bar, 100 μm (right) or 20 μm (magnified image on the left). (C) Two siRNAs (si β‐ca#1, #2) against FAM134B effectively decreased β‐catenin expression in FAM134B‐transfected Bel‐7402 cell, as detected by western blot analysis. Scrambled siRNA (si NC) and GAPDH were used as negative and loading controls, respectively. Cell growth (D) and foci formation (E) were inhibited by silencing of β‐catenin in FAM134B‐transfected Bel‐7402 cells. The results are expressed as the mean ± SEM of three independent experiments. Two‐way ANOVA was used in panel (D), One‐way ANOVA was used in panel (E).

Immunohistochemistry staining was used to determine the expression patterns of β‐catenin and cyclin D1 in xenograft tumors that developed from the FAM134B‐knockdown cells (Fig. 4A) and FAM134B‐transfected cells (Fig. 4B). The expression levels of β‐catenin and cyclin D1 were decreased by FAM134B knockdown, but increased by FAM134B transfection, which is consistent with the results of western blot analysis (Fig. 3A).

Since FAM134B activates the Akt/GSK‐3β/β‐catenin pathway, it was next determined whether this axis was involved in the increase in cell proliferation induced by FAM134B. Briefly, 7402 cells transfected with FAM134B were stably transfected with two siRNAs against β‐catenin, namely 7402 si β‐catenin#1 (FAM si β‐ca#1) and 7402 si β‐catenin#2 (FAM si β‐ca#2), respectively. Scrambled siRNA‐transfected cells (si NC) were used as negative controls. The results showed that si β‐catenin reversed the upregulation of cyclin D1 caused by FAM134B transfection (Fig. 4C). Also, silencing of β‐catenin can reverse the upregulation in cell growth (Fig. 4D) and foci formation induced by FAM134B (Fig. 4E).

### 3.6 FAM134B promotes motility and metastasis in HCC

**Figure 5:**
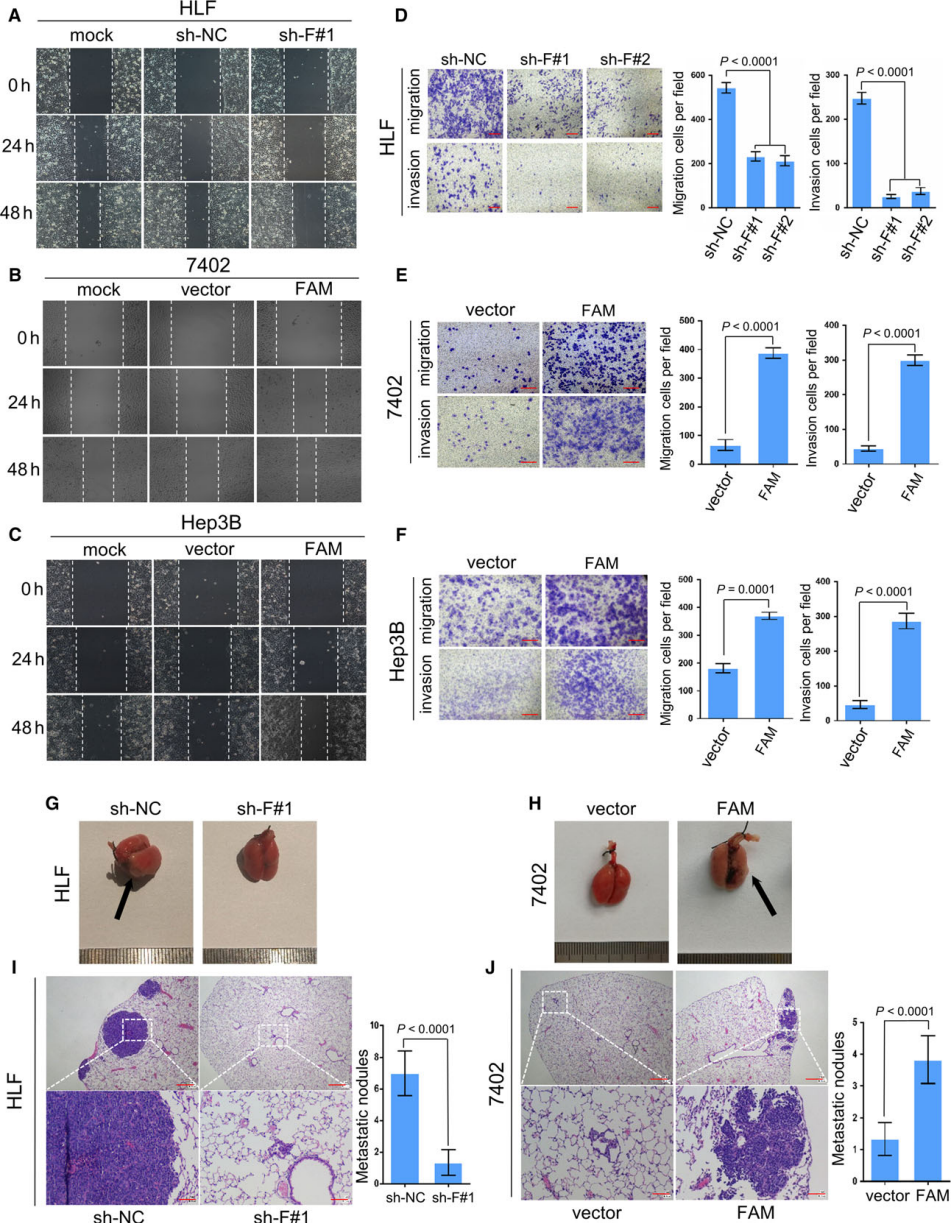
FAM134B promotes motility and metastasis of HCC *in vitro* and *in vivo*. The results of the wound‐healing assay using HLF (A), Bel‐7402 (B), and Hep3B (C) cells showed that FAM134B promoted cell motility. Representative images were taken at 0, 24, and 48 h after scratching. The transwell migration and invasion assay of HLF (D), Bel‐7402 (E), and Hep3B (F) cell showed that FAM134B promoted cell migration and invasion. Representative images of invaded cells are shown in the left panel and the results are summarized in the right panel. The results are expressed as the mean ± SEM of three independent experiments. Scale bar, 100 μm. An experimental metastasis model was used to evaluate the effects of FAM134B on tumor metastasis by tail vein injection of cells *in vivo*. Representative images of lungs derived from nude mice injected with FAM134B‐knockdown (G) and FAM134B‐transfected (H) cells are shown. Scrambled shRNA‐transfected HLF cells and empty vector‐transfected Bel‐7402 cells were used as negative controls. Metastatic nodules on the lung surface are indicated by arrows. Representative images of H&E stained sections derived from the FAM134B‐knockdown (I) and FAM134B‐transfected (J) lung metastatic nodules. Formation of metastatic nodules in the lung are summarized as the mean ± SEM in the right panel. Sections of lung derived from nude mice injected with the indicated cells through tail vein. Scrambled shRNA‐transfected HLF cells and empty vector‐transfected Bel‐7402 cells were used as controls. Scale bar, 500 μm (upper panel) or 100 μm (lower panel). One‐way ANOVA was used in panel (D), Independent Student's *t*‐test was used in panel (E, F, I, and J).

Since FAM134B has strong tumorigenesis features and activates the Akt/GSK‐3β/β‐catenin axis, the role of FAM134B in tumor cell motility and metastasis were investigated. The results of the wound healing assay revealed that wound closure was slower in HLF cells after FAM134B‐knockdown, as compared with control cells (Figs 5A and S5A), while the healing rate of 7402 and Hep3B cells was increased after FAM134B transfection, as compared with the control cells (Figs 5B,C and S5B,C). The transwell migration and invasion assay further showed that FAM134B knockdown significantly decreased migration and invasion of HLF cells (*P *<* *0.0001, Fig. 5D), but increased migration and invasion of FAM134B‐transfected 7402 (*P* < 0.0001, Fig. 5E) and Hep3B (*P *<* *0.001, Fig. 5F) cells. To evaluate the effects of FAM134B on tumor metastasis *in vivo*, four groups of five mice each were injected with HLF sh‐NC and HLF sh‐FAM134B#1, as well as 7402‐vector and 7402‐FAM134B cells into the tail vein, respectively. The mice were sacrificed after 8 weeks (Fig. 5G,H). H&E staining was performed to count the number of lung nodules and to confirm that these were indeed metastatic tumors. A significantly lower number of metastatic nodules were found in lungs of mice injected with the HLF sh‐FAM134B#1 cells than those injected with the HLF sh‐NC cells (*P *<* *0.0001, Fig. 5I), while the number of metastatic nodules caused by injection with 7402‐FAM134B cells injected was much larger than injection with cells carrying the 7402 vector (*P *<* *0.0001, Fig. 5J).

### 3.7 FAM134B induces EMT via Akt activation and Snail expression

**Figure 6:**
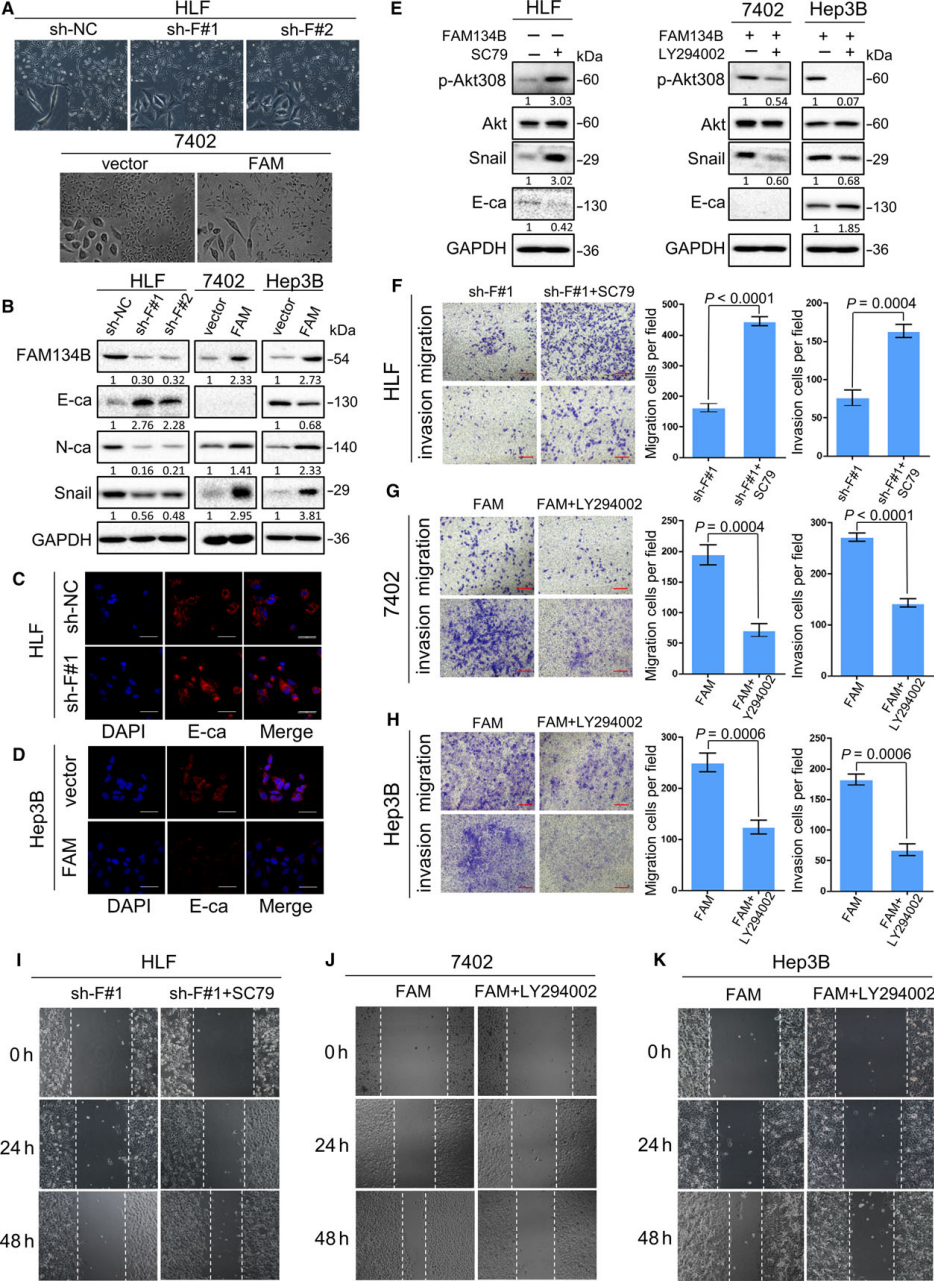
FAM134B regulates E‐cadherin expression through Akt activation and Snail expression to induce EMT. (A) Morphological analyses in stable FAM134B‐knockdown HLF cells as well as stable FAM134B‐transfected Bel‐7402 cells (original magnification, ×100). (B) Western blot analysis comparing relative expression levels of FAM134B, E‐cadherin, N‐cadherin and Snail in FAM134B‐transfected and FAM134B‐ knockdown cells with respective control cells. GAPDH was used as loading control. Representative IF images showing increased expression of E‐cadherin in sh‐FAM134B HLF cells (C) and decreased expression of E‐cadherin in FAM134B‐transfected Hep3B cells (D), as compared with sh‐NC HLF cells. Nuclei were counterstained with 4′,6‐diamidino‐2‐phenylindole. Scale bar, 50 μm. (E) Western blot analysis comparing relative expression levels of phosphorylated Akt, Akt, E‐cadherin, and Snail in FAM134B‐transfected cells treated with LY294002 (10 μm) and FAM134B‐knockdown cells treated with SC79 (10 μm) with respective control cells. GAPDH was used as loading control. Transwell migration and invasion assay of HLF (F), Bel‐7402 (G), and Hep3B (H) cell lines treated with LY294002 and SC79 respectively showing that activation of the Akt signaling pathway promoted cell migration and invasion. Representative images of invaded cells are shown in the left panel and the results are summarized by independent Student's *t*‐test in the right panel. The results are expressed as the mean ± SEM of three independent experiments. Scale bar, 100 μm. Wound‐healing assay of the HLF (I), Bel‐7402 (J), and Hep3B (K) cell lines showing that activation of the Akt signaling pathway promoted cell motility. Representative images were taken at 0, 24, and 48 h after scratching.

As the EMT is a key event in tumor metastasis (Lamouille *et al*., 2014), the role of FAM134B during EMT was investigated. Monitoring of the morphological changes of HLF cells after FAM134B knockdown showed that the cells exhibited an epithelial cell morphology, while the control cells maintained a spindle‐like mesenchymal cell morphology. Correspondingly, Bel‐7402 also exhibited a spindle‐like mesenchymal cell morphology after FAM134B transfection (Fig. 6A). A further investigation of the effect of FAM134B knockdown on EMT marker proteins showed that E‐cadherin and occludin were upregulated, while Snail, vimentin and N‐cadherin were downregulated in HLF cells after FAM134B‐knockdown (Figs 6B and S6). An opposite expression pattern of these proteins was observed in FAM134B‐transfected cells (Figs 6B and S6). Similar results were found at the mRNA level of Snail, indicating that FAM134B affected the transcription of EMT marker proteins (Fig. S7). As the expression of E‐cadherin in 7402 cells was undetectable by western blot analysis, the Hep3B cell line was chosen to investigate whether E‐cadherin expression was affected by FAM134B. IF staining showed that E‐cadherin expression was upregulated in HLF cells after FAM134B‐knockdown, (Fig. 6C) and it was downregulated in FAM134B‐transfected Hep3B cells (Fig. 6D).

Clinical data also showed a direct role of FAM134B in EMT. We detected the expression of E‐cadherin in the 122 tissue microarray of primary HCC tissues (Fig. S8).The association between FAM134B levels and E‐cadherin level in HCC tissues was shown (Table 2). If the IHC scoring of E‐cadherin is higher in the tumor tissue than in the adjacent non‐tumor tissue, we defined it as Positive. On the contrary, if the IHC scoring of E‐cadherin is lower in the tumor tissue than in the adjacent non‐tumor tissue, we defined it as Negative. Results showed that the correlation coefficient between FAM134B levels and E‐cadherin levels was 0.304. And the expression of E‐cadherin was relatively low in 76.8% (43/56) of FAM134B high expression HCC tissues, compared with 46.9% (31/66) of FAM134B non‐high expression HCC tissues. IHC staining on xenograft tumors also showed that the expression of E‐cadherin is relatively high after FAM134B was knockdown (Fig. S9).

**Table 2:** Crosstable of FAM134B expression and E‐cadherin expression

	FAM134B by IHC			*R*	*P*
	Total	Non‐high expression	High expression		
E‐cadherin by IHC					
Total	122	66	56		
Negative	74	31	43	0.304	0.001
Positive	48	35	13		

These results demonstrated that the activation of Akt acts a pivotal part in inducing tumorigenesis by inhibiting GSK‐3β earlier, which leads to the accumulation of β‐catenin. In the mechanism of metastasis induced by FAM134B, decreasing GSK‐3β expression led to the accumulation of Snail, but it remained unclear whether Akt activated by FAM134B triggers EMT. Hence, the Akt activator SC79 and the PI3K inhibitor LY294002 were used to determine if there was a correlation between Akt expression and FAM134B‐induced cell metastasis. The results revealed that SC79 increased the expression levels of Snail downregulated by FAM134B knockdown in HLF cells (Fig. 6E), while LY294002 decreased the expression levels of Snail upregulated by FAM134B transfection of 7402 and Hep3B cells (Fig. 6E). SC79 reversed the ability of cell migration, invasion (Fig. 6F), and motility (Figs 6I and S5D), which decreased after FAM134B knockdown in HLF cells, while LY294002 also reversed the ability of cell migration, invasion (Fig. 6G,H), and motility (Figs 6J,K and S5E,F), which was acquired in 7402 and Hep3B cells following FAM134B transfection.

### 3.8 FAM134B promotes cell metastasis by Snail stabilization

**Figure 7:**
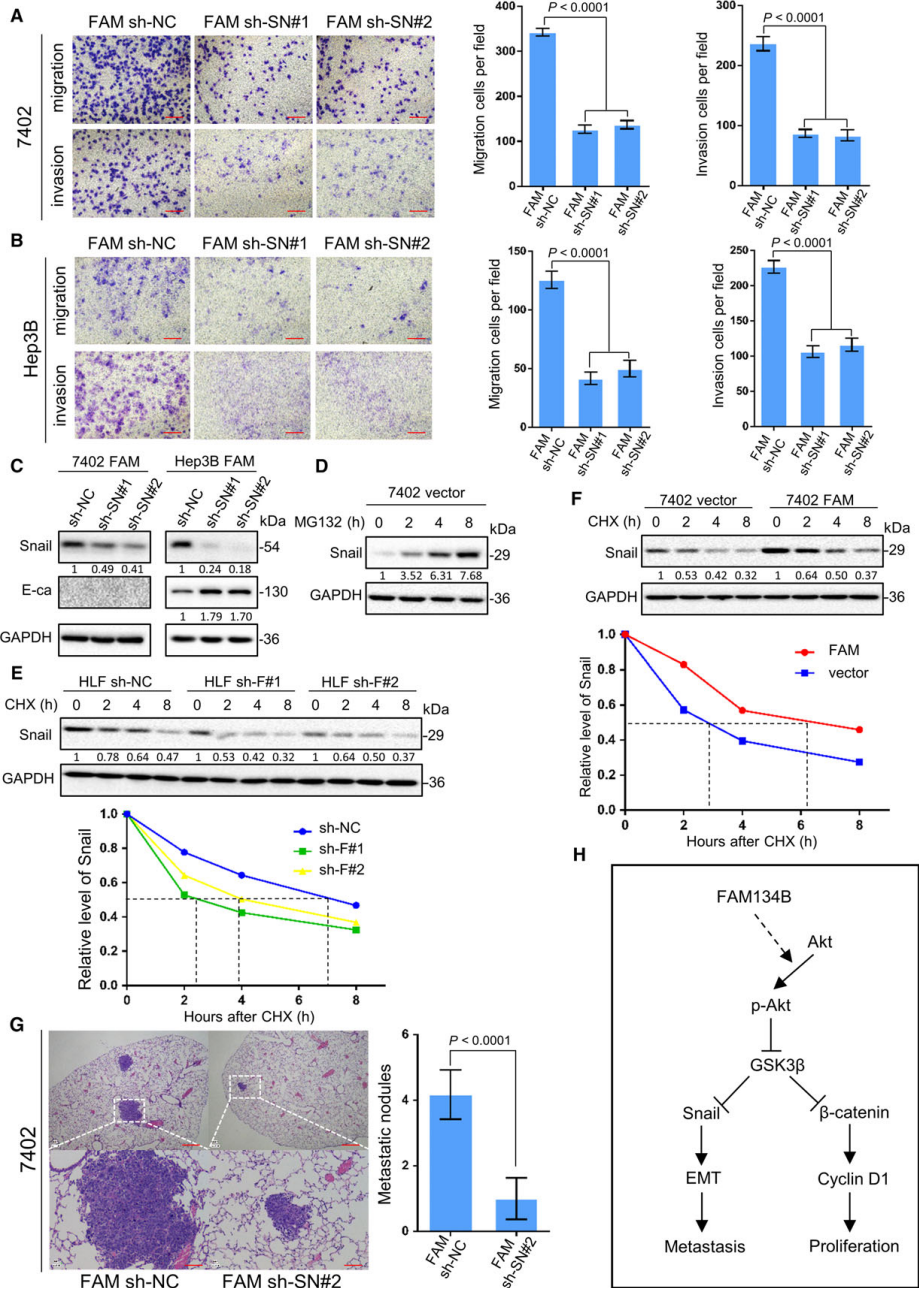
FAM134B promotes cell metastasis by Snail stabilization. Two shRNAs (sh β‐ca#1, #2) against Snail inhibited migration and invasion of FAM134B‐transfected Bel‐7402 cells (A) and Hep3B (B) cells, as detected by the transwell assay, the results are expressed as the mean ± SEM of three independent experiments by One‐way ANOVA. Scale bar, 100 μm. (C) The expression of E‐cadherin was increased in FAM134B‐transfected Hep3B cells, as detected by western blot analysis. Scrambled shRNA (sh NC) and GAPDH were used as negative and loading controls, respectively. (D) Snail in Bel‐7402 cells was degraded permanently. Cells were treated with MG132 (10 μm) for the indicated periods and then harvested for IF analysis. GAPDH was used as loading control (E) FAM134B knockdown shortened the half‐life of Snail. Cells were treated with cycloheximide for the indicated periods and harvested for IF analysis. (F) Overexpression of FAM134B extended the half‐life of Snail. Cells were treated with cycloheximide for the indicated periods and harvested for IF analysis. (G) FAM134B‐transfected Bel‐7402 cells were transfected with shRNA targeting Snail. An experimental metastasis model was used to evaluate the effects of Snail *in vivo* on tumor metastasis by tail vein injection of cells. Representative images of H&E‐stained sections derived from the FAM134B‐knockdown and FAM134B‐transfected with Snail knockdown lung metastatic nodules Scale bar, 500 μm (upper panel) or 100 μm (lower panel). Formation of metastatic nodules in the lung are summarized as the mean ± SEM in the right panel by independent Student's *t*‐test. (H) Schematic diagram representing the role of FAM134B in tumorigenesis and EMT in HCC.

Snail is a crucial transcription factor that regulates the expression of E‐cadherin during EMT (Grande *et al*., 2015; Lamouille *et al*., 2014). To investigate whether Snail is of vital importance in EMT induced by FAM134B, FAM134B‐transfected 7402 and Hep3B cells were stably transfected with two shRNAs sequences against Snail, namely FAM134B‐transfected sh‐Snail#1 (FAM sh‐SN#1) and FAM134B‐transfected sh‐Snail#2 (FAM sh‐SN#2), respectively. Scrambled shRNA‐transfected cells (FAM sh‐NC) were used as negative controls. The results showed that Snail knockdown reversed the migration and invasion of 7402 and Hep3B cells promoted by FAM134B transfection (Fig. 7A,B, respectively). Also, Snail knockdown increased the expression of E‐cadherin following FAM134B transfection of 7402 and Hep3B cells (Fig. 7C).

Next, use of the proteasome inhibitor MG132 to inhibit the degradation of Snail showed that the amount of Snail had accumulated sharply in cells transfected with the 7402 vector (Fig. 7D), indicating that Snail is constantly degraded, while Snail is synthesized and finally dynamically balanced in cells transfection of 7402 cells.

Then, the effect of FAM134B on the stability of the Snail protein was investigated. HLF and 7402 cells were treated with cycloheximide to block protein biosynthesis. The results revealed that after FAM134B knockdown, the half‐life of Snail was shortened from 7 to 2.2 or 4 h in HLF cells (Fig. 7F), but prolonged from 3 to 6 h in 7402 cells (Fig. 7E). Comparable results were generated using cycloheximide. These results demonstrated that FAM134B stabilized Snail expression in HCC cells.

To evaluate the *in vivo* effects of Snail on tumor metastasis induced by FAM134B, two groups of five mice each were injected intravenously in the tail vein with 7402 FAM134B‐transfected sh‐NC cells and 7402 FAM134B‐transfected sh‐Snail#2 cells, respectively. After 8 weeks, the mice were sacrificed and the numbers of metastatic nodules in the lungs were counted. H&E staining confirmed that the lung nodules were metastatic tumors. A significantly decreased number of metastatic nodules were induced in lungs of mice injected with the 7402 FAM134B‐transfected sh‐Snail#2 cells, as compared to control cells (*P *<* *0.0001, Fig. 7G).

## 4 Discussion

In this study, the oncogenicity of FAM134B and its mechanism in HCC were investigated. The *Fam134b* gene has been reported to be a frequently amplified regions in gastric carcinoma (Bi *et al*., 2009). The results of the present study showed that FAM134B was overexpressed in 62% (*n* = 50) of HCC patients as well as 45.9% (56/122) of HCC tumor tissue microarray, which was associated with increased tumor size (*P* = 0.025), pathological vascular invasion (*P* = 0.026), lower differentiation grade (*P* = 0.023), cancer recurrence (*P* = 0.044) and portal vein tumor thrombus (*P* = 0.036) in HCC. Furthermore, overexpression of FAM134B predicts a worse prognosis and shorter survival in HCC patients. The results of the functional studies revealed that FAM134B had great tumorigenicity, which could promote cell growth, migration, and invasion in cell lines, as well as tumor formation in nude mice. These effects were effectively inhibited by FAM134B knockdown with shRNA.

Cell proliferation is usually related to apoptosis and/or cell cycle. However, knockdown of FAM134B in HCC cells led to G1 phase arrest rather than apoptosis. The number of cells in G1 phase was decreased by overexpression of FAM134B. Cyclin D1 regulated cell cycle from the G1 phase into the S phase (Takahashiyanaga and Sasaguri, 2008). Upstream activation of GSK‐3β promoted the degradation of cyclin D1 (Diehl *et al*., 1998; Takahashiyanaga and Sasaguri, 2008). Activation of the Akt signaling pathway induced the phosphorylation of GSK‐3β (Cohen and Frame, 2001; Manning and Toker, 2017) and inactivated GSK‐3β, thereby reducing phosphorylation of β‐catenin, which led to accumulation of β‐catenin (Mao *et al*., 2009). β‐catenin can activate lymphoid enhancing factor‐1 (LEF‐1) at the transcription level. The cyclin D1 promoter has a LEF‐1 binding site and upregulation of β‐catenin causes an increase in cyclin D1 expression, which promotes the proliferation of HCC cells (Mosimann *et al*., 2009). We concluded that the expression of phosphorylated Akt increased or decreased accordingly with the overexpression or knockdown of FAM134B, respectively. This change was positively correlated with the expression levels of phosphorylated GSK‐3β and β‐catenin. Therefore, these results demonstrated that FAM134B promoted the proliferation in HCC cells via the Akt/GSK‐3β/β‐catenin signaling pathway (Fig. 7H).

FAM134B promotion of tumor metastasis is related to EMT, which plays a vital part role in tumor metastasis. Hence, we investigated whether the changes in cell motility and metastasis were associated to the EMT process by FAM134B. FAM134B transfection caused a increased the expression of the EMT transcriptional factor Snail and decrease in the protein levels of the epithelial marker E‐cadherin, which confirmed our assumption. Snail is the key point of EMT triggered by FAM134B. E‐cadherin, regulated by Snail, is an epithelial marker to reflect the EMT process. Occludin, an epithelial marker, also acts as the downstream molecule of Snail (Ikenouchi *et al*., 2003). Since E‐cadherin is undetectable in Bel‐7402 cell line, we detected the expression of occludin (Fig. S6) instead of E‐cadherin in Bel‐7402 cell line.

Snail is an important EMT transcription factor (Nieto *et al*., 2016) that was modulated by FAM134B expression. FAM134B increases Snail mRNA levels and also inhibit the degradation of Snail, thereby extending its half‐life. As a downstream target of Akt, GSK‐3β phosphorylated Snail and induced its degradation and nuclear export (Zhou *et al*., 2004). Akt activation promoted GSK‐3β phosphorylation and subsequent dysfunction, thereby triggering EMT of HCC cells. Taken together, these findings demonstrated that FAM134B promoted EMT through the Akt/GSK‐3β/Snail signaling pathway (Fig. 7H).

Despite the functions in tumor biology, FAM134B also has some other features. In the normal physiological state, FAM134B protein is involved in the homeostasis of cells by regulating endoplasmic reticulum (ER)‐turnover. FAM134B protein serves as a receptor for the components of ER‐autophagic machinery (ER‐phagy machinery) and regulates the continuous turnover of ER in cells (Khaminets *et al*., 2015). A recent study demonstrated that FAM134B restricted the replication of Dengue, Zika and West Nile virus at the early stage of the viral life cycle (Lennemann and Coyne, 2017). We believed one of the reasons is that ER phagy induced by FAM134B constitutively functions as the ER‐quality control to remove overexpressed viral proteins. Nevertheless, mutations of FAM134B with loss‐of‐function of the gene leads to hereditary autonomic neuropathies IIB in human, which is also related to the ER‐turnover induced by FAM134B (Ilgaz Aydinlar *et al*., 2014; Kurth *et al*., 2009; Murphy *et al*., 2012). Since FAM134B protein regulates ER turnover, overexpression/downregulation of FAM134B could affect the processing and/or transport of molecules which are involved in pluripotency. Thus, this function of FAM134B implicates that it may have a role in controlling stemness (Islam *et al*., 2017).

In summary, *Fam134b* is a novel oncogene in HCC and acts as a critical role in the tumorigenesis and metastasis of HCC cells. Our study shows that FAM134B activates the Akt signaling pathway and mediates downstream carcinogenic effects. Due to the ubiquity of FAM134B in normal mammalian cells, its function remains to be further studied, including the opposite effect on carcinogenesis in different organs and the critical point of maintaining ER turnover in the promotion of HCC onset, especially the interaction with the Akt signaling pathway. Once these mechanisms are well investigated, FAM134B is promising to be a new diagnostic or therapeutic target for HCC.

## 5 Conclusion

We demonstrated that FAM134B enhanced Akt activity with subsequent GSK‐3β phosphorylation, accumulation of β‐catenin, and stabilization of Snail, which promoted tumorigenesis, EMT, and tumor metastasis in HCC by experiments *in vitro* and *in vivo*.

## Authors contributions

ZqZ and JC conceived and designed the project. ZqZ, JC, WqH and DN performed the experiments. QmL, CW and LZ collected the clinical specimens and data. ZqZ and WqH participated performed the statistical analysis and wrote the manuscript. LR, LC, HfL, BxZ and CpC contributed to the writing and to the critical reading of the paper. All authors read and approved the final manuscript. All authors read and gave their approval for the final version of the manuscript.

## Conflicts of interest

The authors have no conflicts of interest to declare.

## Supporting information


**Fig. S1**. H&E stain in xenograft tumors.

**Fig. S2**. The canonical signaling pathway affected by FAM134B.

**Fig. S3**. Erk signaling pathway is not involved in HCC proliferation induced by FAM134B.

**Fig. S4**. FAM134B did not affect apoptosis of HCC cells.

**Fig. S5**. Quantitative analysis for the results of healing assay.

**Fig. S6**. FAM134B regulates the expression of EMT markers.

**Fig. S7**. FAM134B can affect the expression of Snail at the transcription level.

**Fig. S8**. IHC analysis of E‐cadherin and FAM134B expression in 122 paired HCC tissues.

**Fig. S9**. E‐cadherin is upregulated after FAM134B was knockdown in the IHC of xenograft tumors.
