# Supplementary figures and images for: FAM134B induces tumorigenesis and epithelial‐to‐mesenchymal transition via Akt signaling in hepatocellular carcinoma
Source: Mol Oncol. 2019 Jan 24;13(4):792–810. doi: 10.1002/1878-0261.12429 (PMC6441892; doi:10.1002/1878-0261.12429)

## Slide 1
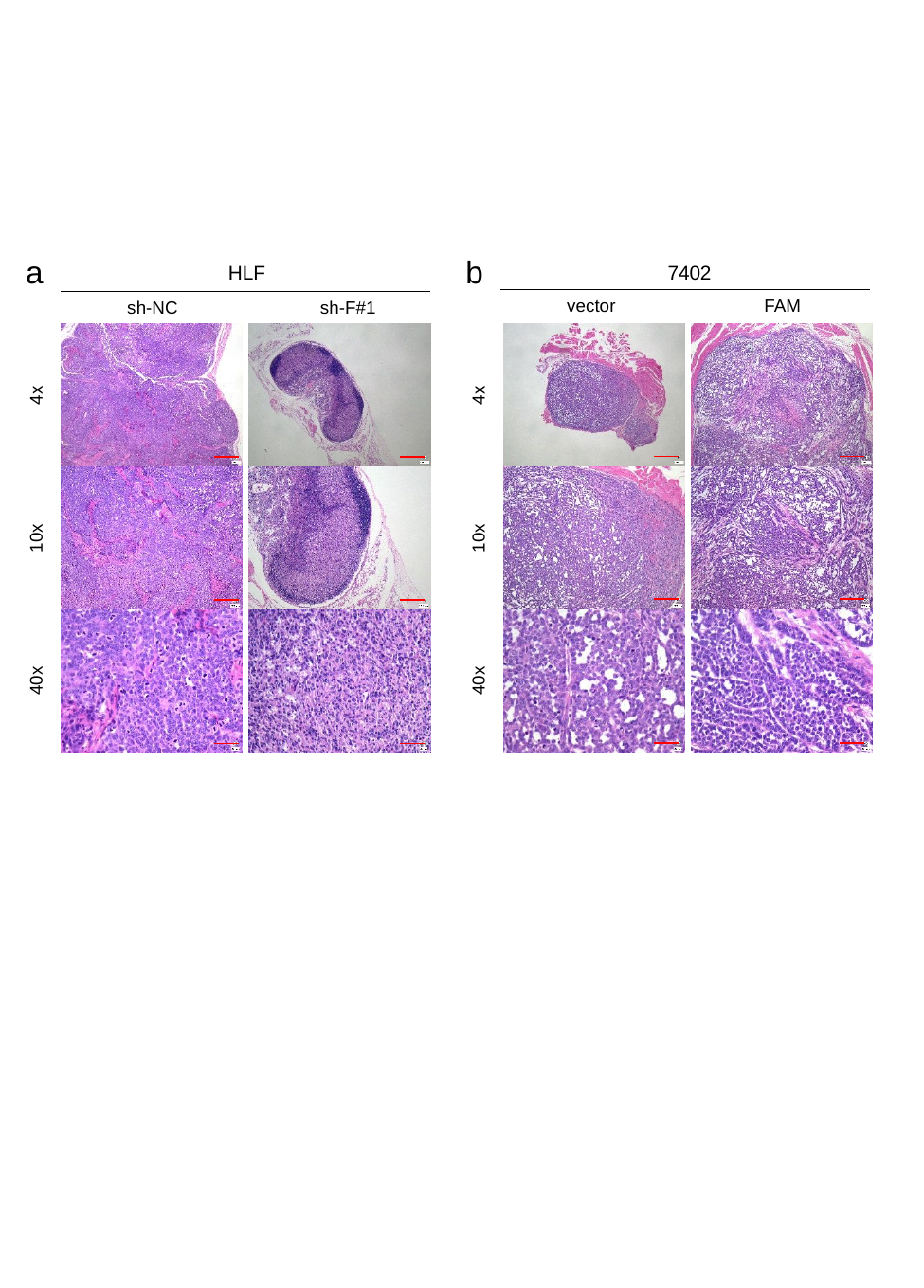

a
b
7402
vector
FAM
HLF
sh-NC
sh-F#1
4x
4x
10x
10x
40x
40x

Supplement: Supplementary file 1 — Fig. S1. H&E stain in xenograft tumors. [file MOL2-13-792-s001.pptx]

## Slide 1
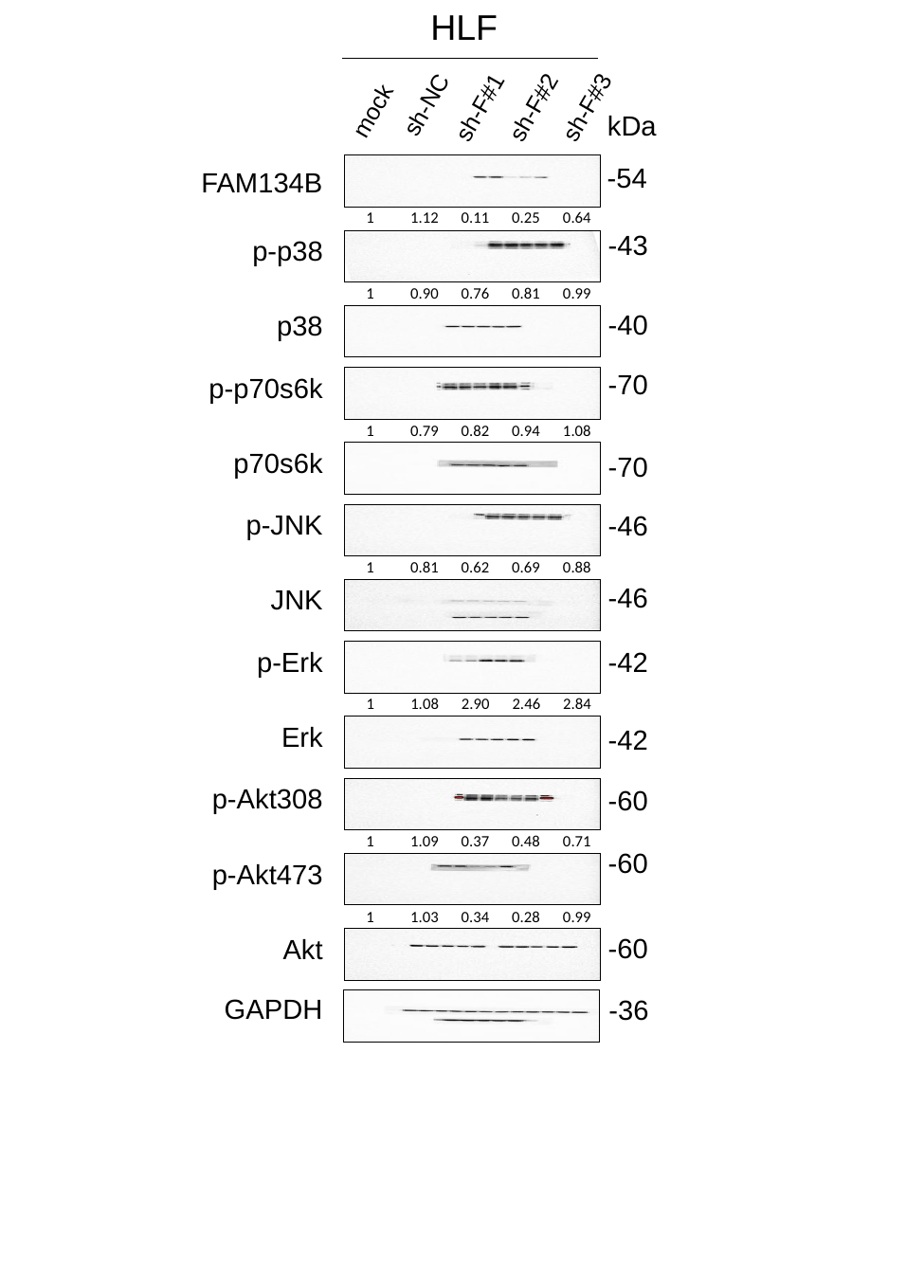

HLF
sh-NC
sh-F#1
sh-F#2
sh-F#3
mock
kDa
-54
FAM134B
1
1.12
0.11
0.25
0.64
-43
p-p38
1
0.90
0.76
0.81
0.99
-40
p38
-70
p-p70s6k
1
0.79
0.82
0.94
1.08
p70s6k
-70
p-JNK
-46
1
0.81
0.62
0.69
0.88
-46
JNK
p-Erk
-42
1
1.08
2.90
2.46
2.84
Erk
-42
p-Akt308
-60
1
1.09
0.37
0.48
0.71
-60
p-Akt473
1
1.03
0.34
0.28
0.99
-60
Akt
GAPDH
-36

Supplement: Supplementary file 2 — Fig. S2. The canonical signaling pathway affected by FAM134B. [file MOL2-13-792-s002.pptx]

## Slide 1
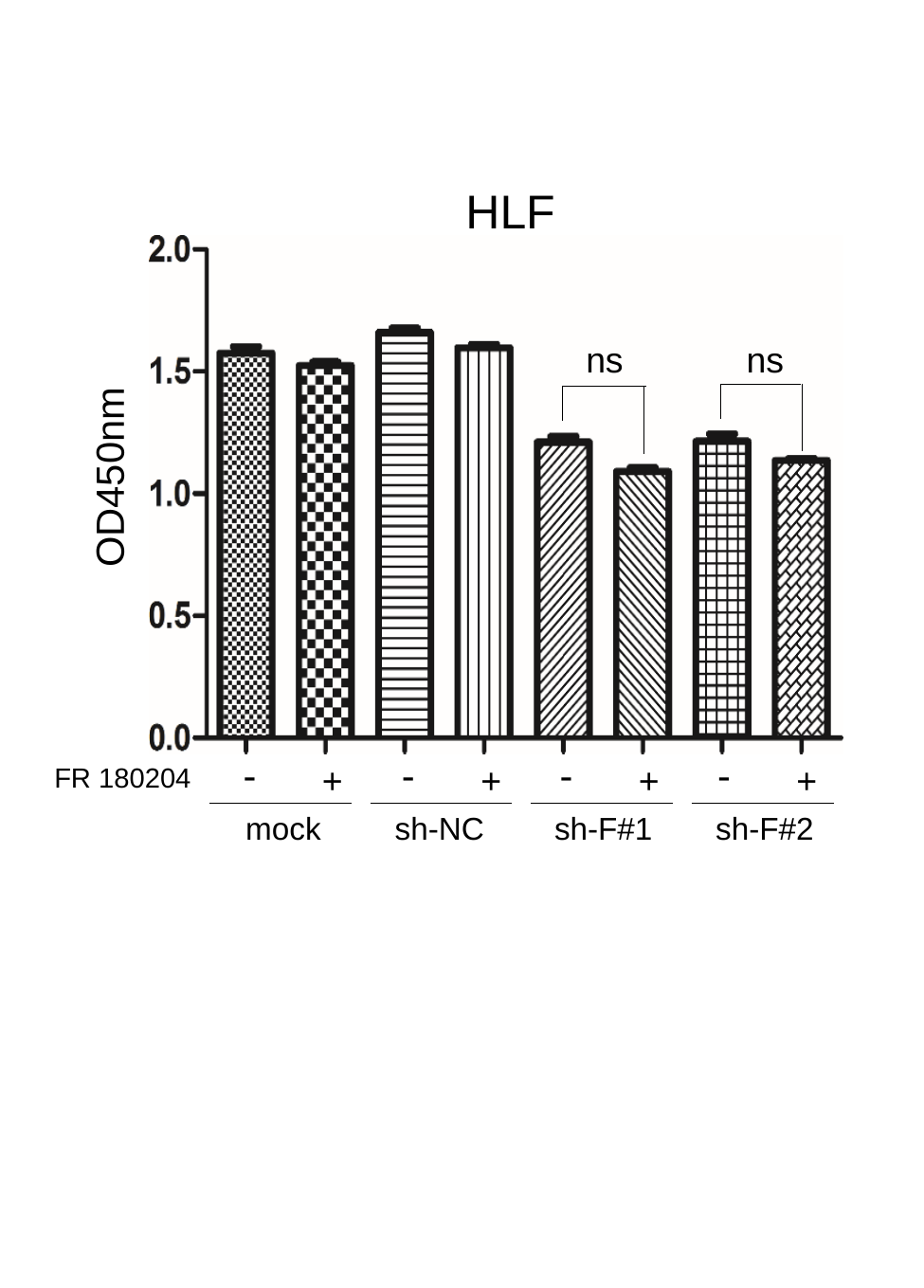

HLF
OD450nm
-
+
-
+
-
+
-
+
FR 180204
mock
sh-NC
sh-F#1
sh-F#2
ns
ns

Supplement: Supplementary file 3 — Fig. S3. Erk signaling pathway is not involved in HCC proliferation induced by FAM134B. [file MOL2-13-792-s003.pptx]

## Slide 1
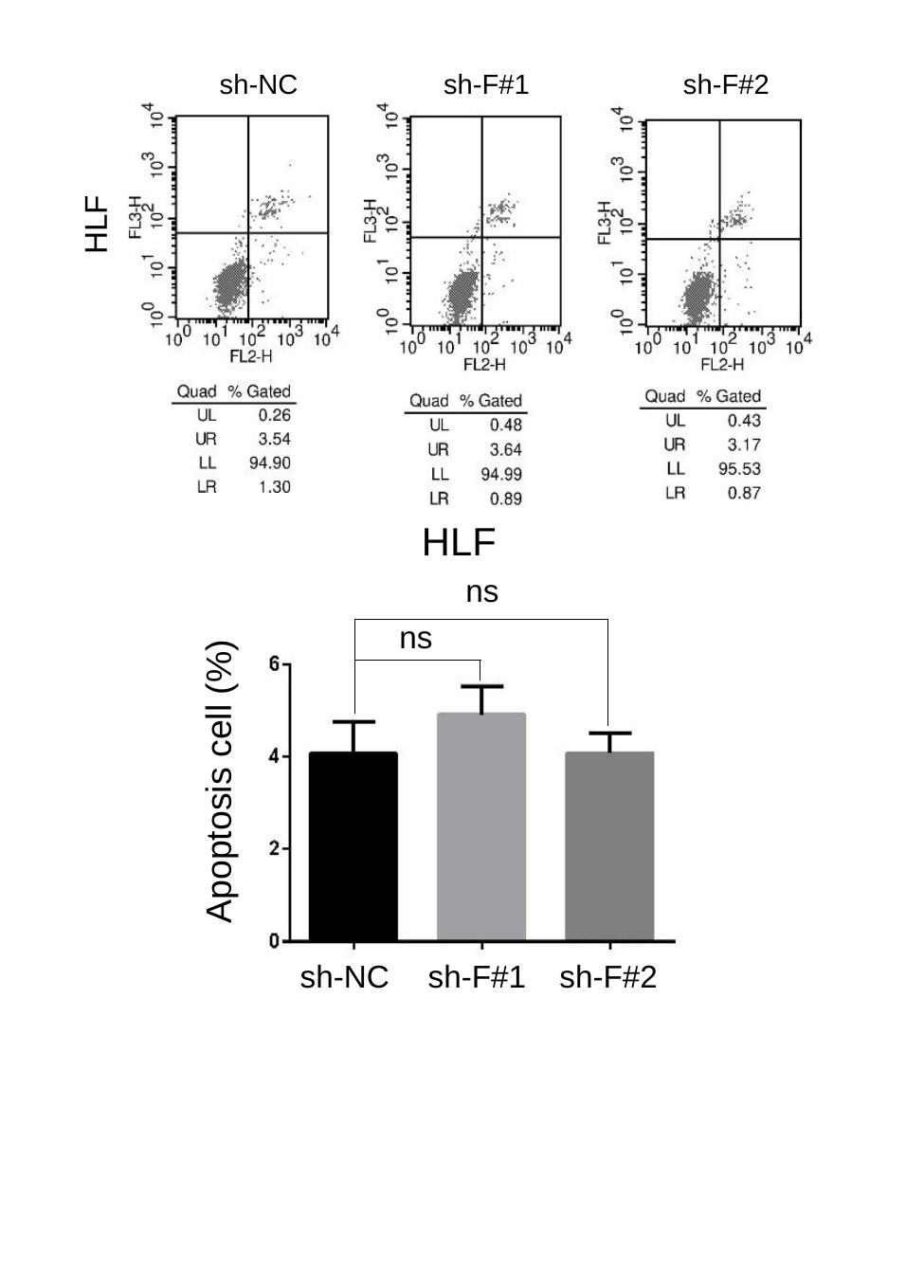

sh-NC
sh-F#1
sh-F#2
HLF
HLF
Apoptosis cell (%)
ns
ns
sh-NC
sh-F#1
sh-F#2

Supplement: Supplementary file 4 — Fig. S4. FAM134B did not affect apoptosis of HCC cells. [file MOL2-13-792-s004.pptx]

## Slide 1
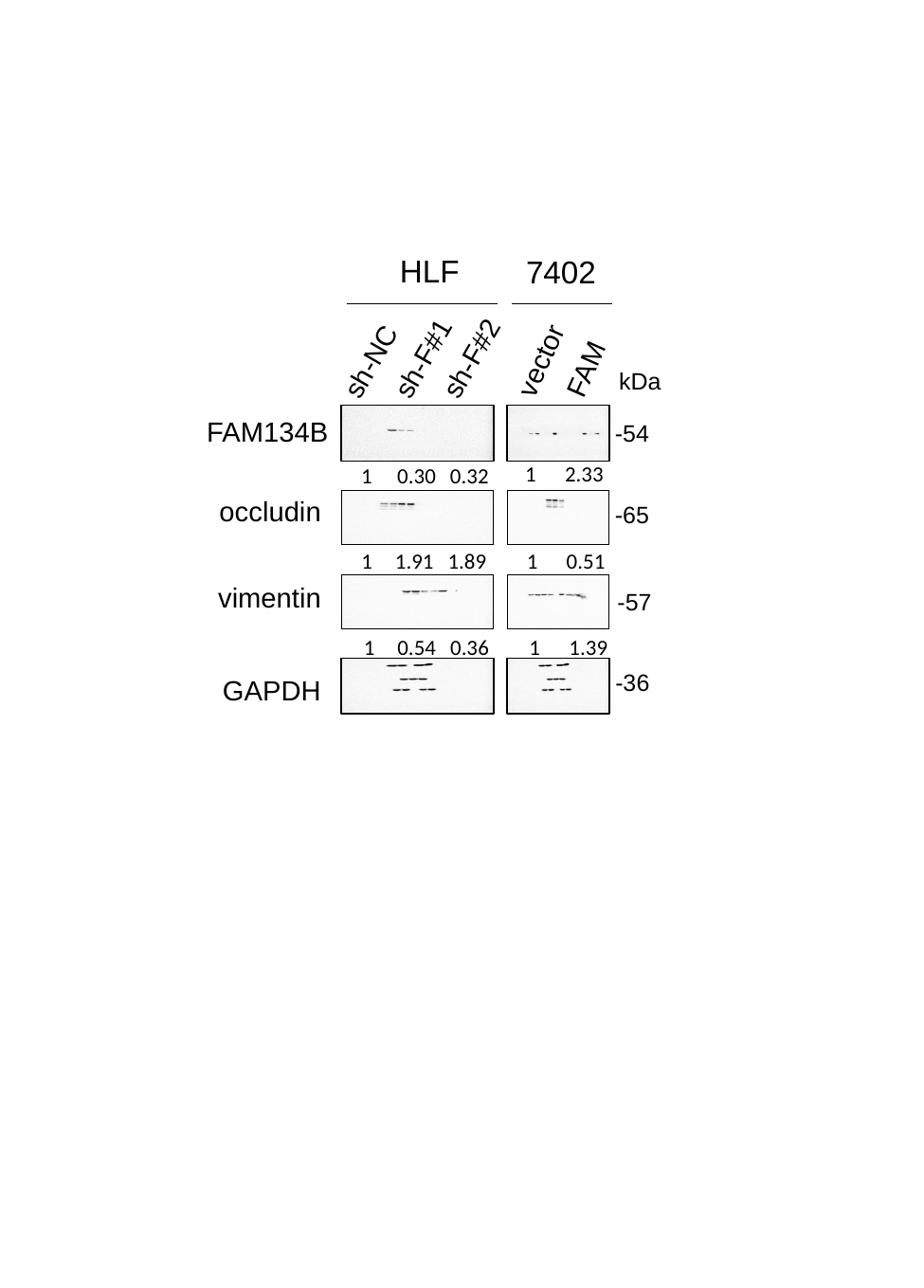

HLF
7402
sh-F#1
sh-F#2
vector
sh-NC
FAM
kDa
FAM134B
-54
1
2.33
1
0.30
0.32
occludin
-65
1
1.91
1.89
1
0.51
vimentin
-57
1
0.54
0.36
1
1.39
-36
GAPDH

Supplement: Supplementary file 6 — Fig. S6. FAM134B regulates the expression of EMT markers. [file MOL2-13-792-s006.pptx]

## Slide 1
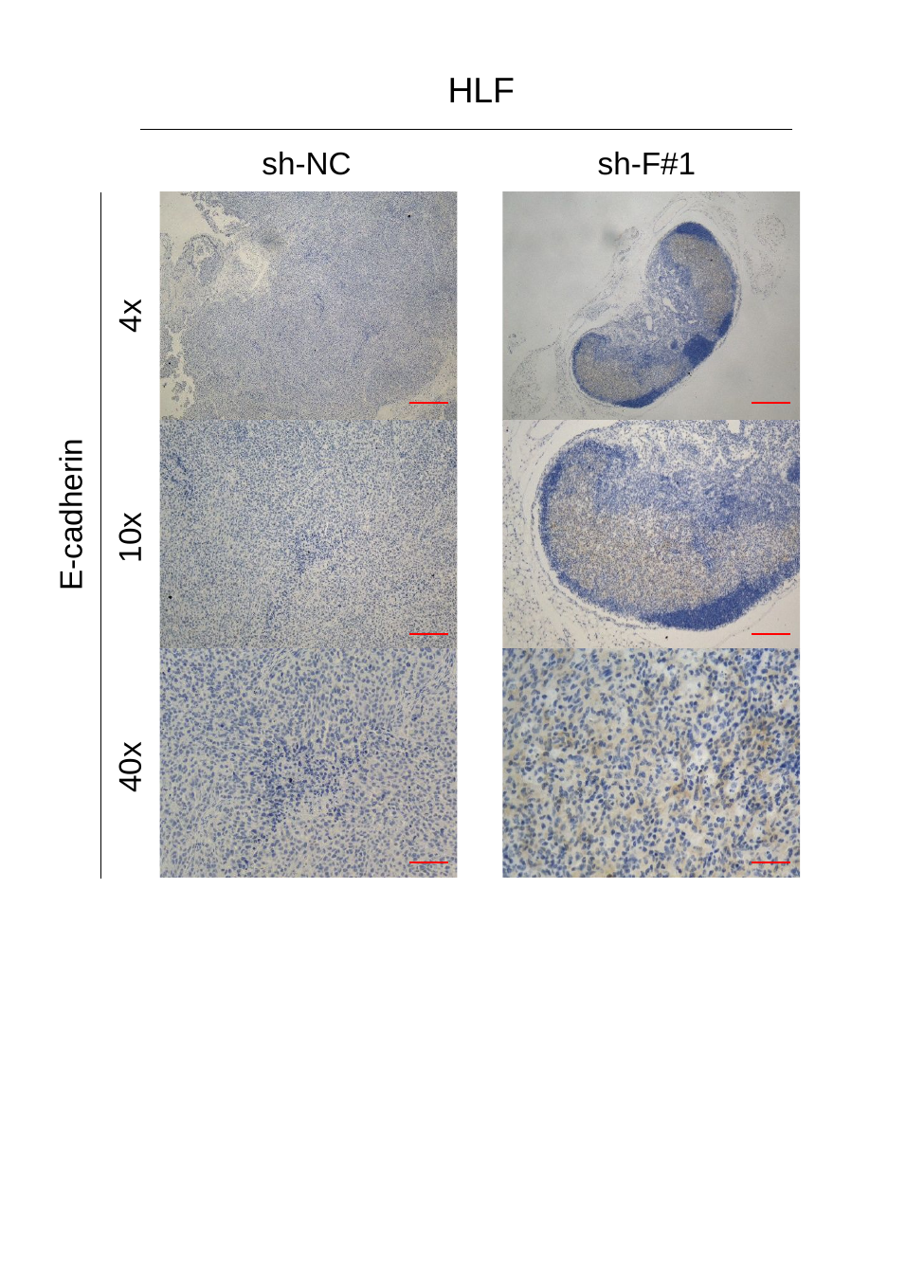

HLF
sh-NC
sh-F#1
4x
E-cadherin
10x
40x

Supplement: Supplementary file 9 — Fig. S9. E‐cadherin is upregulated after FAM134B was knockdown in the IHC of xenograft tumors. [file MOL2-13-792-s009.pptx]
