# Supplementary material for: FAM134B induces tumorigenesis and epithelial‐to‐mesenchymal transition via Akt signaling in hepatocellular carcinoma
Source: Mol Oncol. 2019 Jan 24;13(4):792–810. doi: 10.1002/1878-0261.12429 (PMC6441892; doi:10.1002/1878-0261.12429)
Supplement: Supplementary file 5 — Fig. S5. Quantitative analysis for the results of healing assay. [file MOL2-13-792-s005.pptx]

## Slide 1
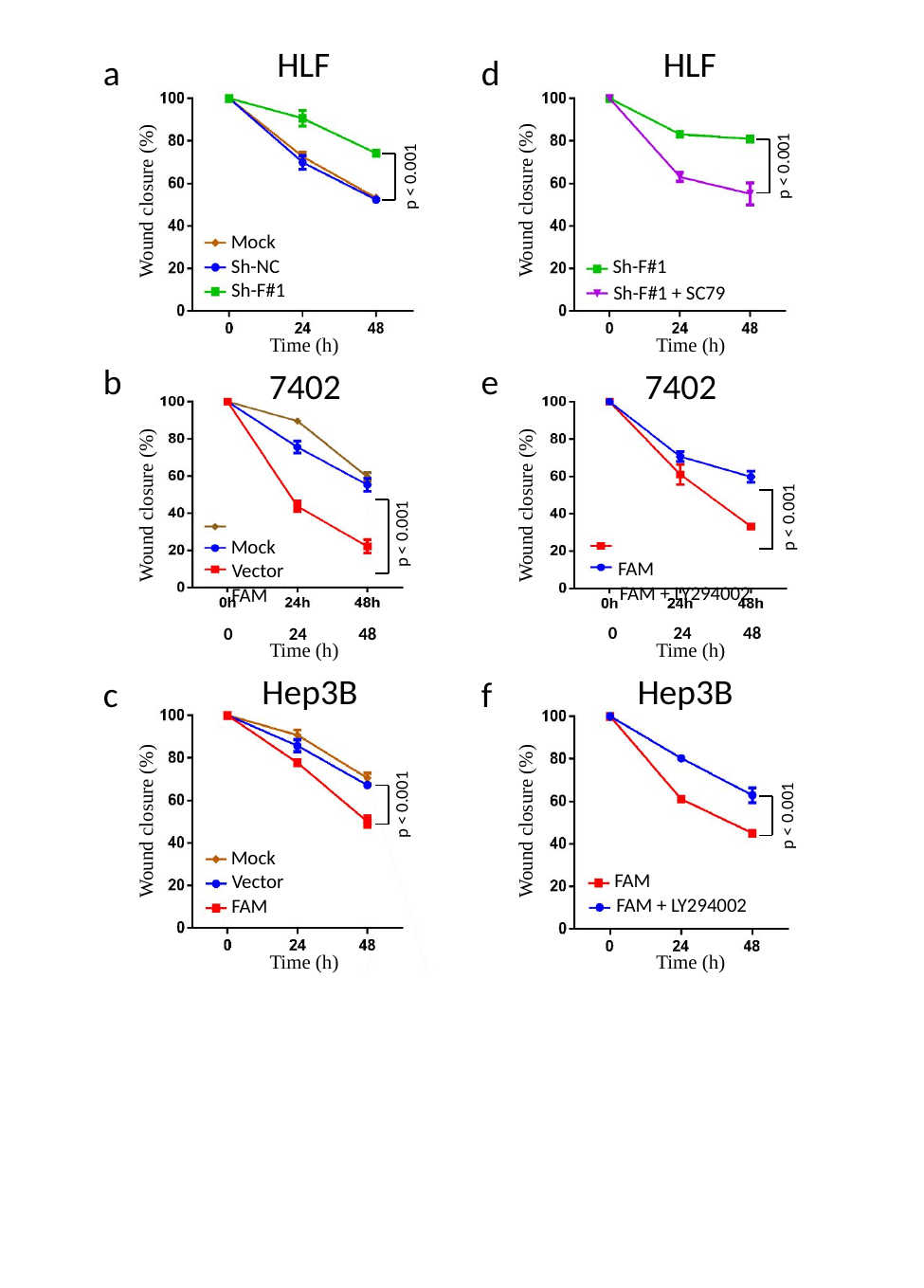

HLF
HLF
a
d
p < 0.001
p < 0.001
Wound closure (%)
Wound closure (%)
Mock
Sh-NC
Sh-F#1
Sh-F#1
Sh-F#1 + SC79
Time (h)
Time (h)
b
e
7402
7402
Wound closure (%)
Wound closure (%)
p < 0.001
p < 0.001
Mock
FAM
Vector
FAM + LY294002
FAM
0
24
48
0
24
48
Time (h)
Time (h)
Hep3B
Hep3B
c
f
p < 0.001
p < 0.001
Wound closure (%)
Wound closure (%)
Mock
FAM
Vector
FAM + LY294002
FAM
Time (h)
Time (h)
