# Supplementary material for: FAM134B induces tumorigenesis and epithelial‐to‐mesenchymal transition via Akt signaling in hepatocellular carcinoma
Source: Mol Oncol. 2019 Jan 24;13(4):792–810. doi: 10.1002/1878-0261.12429 (PMC6441892; doi:10.1002/1878-0261.12429)
Supplement: Supplementary file 7 — Fig. S7. FAM134B can affect the expression of Snail at the transcription level. [file MOL2-13-792-s007.pptx]

## Slide 1
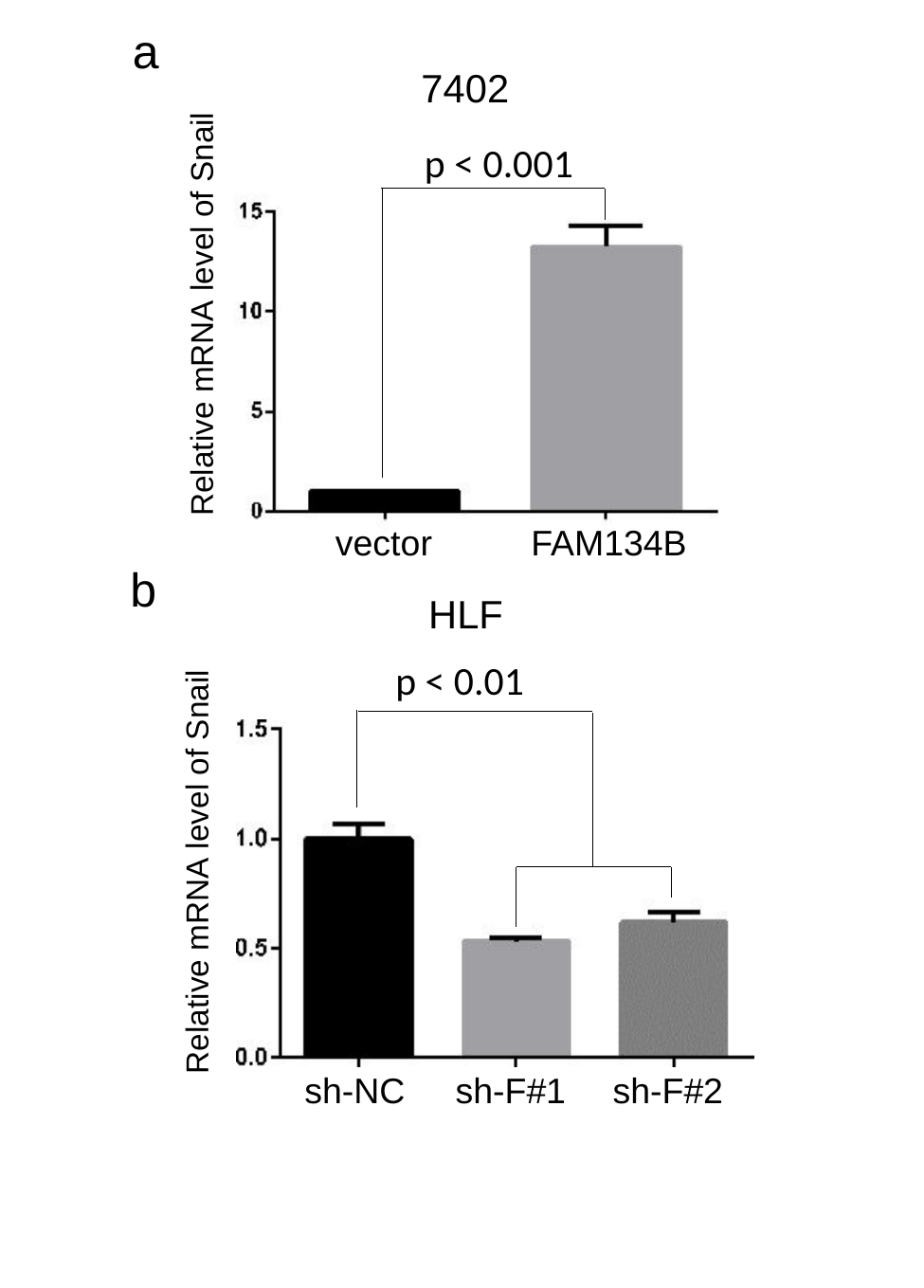

Relative mRNA level of Snail
a
7402
p < 0.001
vector
FAM134B
b
HLF
p < 0.01
sh-NC
sh-F#1
sh-F#2
Relative mRNA level of Snail
