# Supplementary material for: FAM134B induces tumorigenesis and epithelial‐to‐mesenchymal transition via Akt signaling in hepatocellular carcinoma
Source: Mol Oncol. 2019 Jan 24;13(4):792–810. doi: 10.1002/1878-0261.12429 (PMC6441892; doi:10.1002/1878-0261.12429)
Supplement: Supplementary file 8 — Fig. S8. IHC analysis of E‐cadherin and FAM134B expression in 122 paired HCC tissues. [file MOL2-13-792-s008.pptx]

## Slide 1
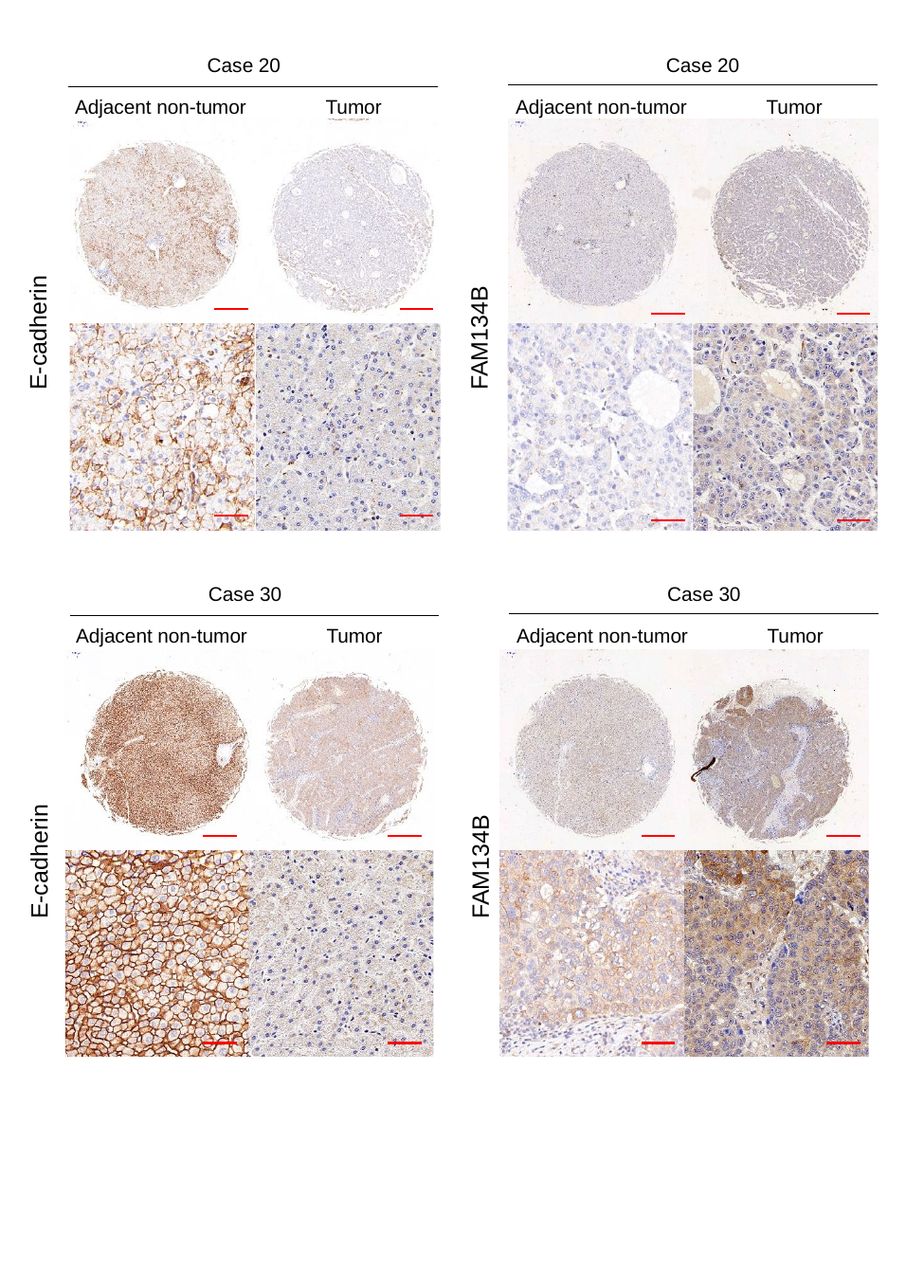

Case 20
Case 20
Adjacent non-tumor
Tumor
Adjacent non-tumor
Tumor
E-cadherin
FAM134B
Case 30
Case 30
Adjacent non-tumor
Tumor
Adjacent non-tumor
Tumor
E-cadherin
FAM134B
