# Supplementary material for: FAM134B induces tumorigenesis and epithelial‐to‐mesenchymal transition via Akt signaling in hepatocellular carcinoma
Source: Mol Oncol. 2019 Jan 24;13(4):792–810. doi: 10.1002/1878-0261.12429 (PMC6441892; doi:10.1002/1878-0261.12429)
Supplement: Supplementary file 10 [file MOL2-13-792-s010.docx]

**Supplementary Figure legends**

**Supplementary Figure 1. H&E stain in xenograft tumors.** Representative images of H&E stained sections derived from the FAM134B-knockdown (a) and FAM134B-transfected (b) xenograft tumors compared with the control group respectively. Scale bar, 500 µm (upper panel), 200 µm (middle panel), or 50 µm (lower panel).

**Supplementary Figure 2. The canonical signaling pathway affected by FAM134B.** Knockdown of FAM134B inhibited the Akt signaling pathway and activated the Erk signaling pathway. Western blot analysis of the relative expression levels of FAM134B, phosphorylated p38, p38, phosphorylated p70s6k, p70s6k, phosphorylated JNK, JNK, phosphorylated Erk, Erk, phosphorylated Akt, and Akt in FAM134B-knockdown cells versus sh-NC scramble control cells. GAPDH was used as a loading control.

**Supplementary Figure 3.** **Erk signaling pathway is not involved in HCC proliferation induced by FAM134B.** The CCK8 assay was used to evaluate the growth of FAM134B-knockdown cells treated with or without FR180204 (10 µM). The results are presented as the mean ± SEM of six independent experiments run in duplication by two-way ANOVA (ns, not significant).

**Supplementary Figure 4. FAM134B did not affect apoptosis of HCC cells.** Flow cytometry analysis of apoptosis of HLF cells with or without FAM134B depletion. The results are presented as the mean ± SEM of triplicate determinations from three independent experiments by one-way ANOVA (ns, not significant).

**Supplementary Figure 5. Quantitative analysis for the results of healing assay.** The quantitative analysis of the wound-healing assay using HLF (a), Bel-7402 (b), and Hep3B (c) cells showed that FAM134B promoted cell motility. The reverse wound-healing assay using HLF (d), Bel-7402 (e), and Hep3B (f) cells showed that activation of the Akt signaling pathway promoted cell motility. The results are expressed as the mean ± SEM of three independent experiments by two-way ANOVA.

**Supplementary Figure** **6. FAM134B regulates the expression of EMT markers.** Western blot analysis comparing relative expression levels of FAM134B, occludin and vimentin in FAM134B-transfected and FAM134B-knockdown cells with respective control cells. GAPDH was used as loading control.

**Supplementary Figure 7. FAM134B can affect the expression of Snail at the transcription level.** mRNA levels of Snail in FAM134B-transfected and FAM134B-knockdown cells were determined by quantitative real-time PCR. Results are presented as the mean ± SEM of six determinations from three independent experiments Independent Student’s *t*-test was used in panel a, while one-way ANOVA was used in panel b.

**Supplementary Figure** **8. IHC analysis of E-cadherin and FAM134B expression in 122 paired HCC tissues.** Representative images of E-cadherin expression in a matched primary HCC sample and its adjacent non-tumor tissue. The expression of FAM134B is shown in the right panel. Scale bar, 300 µm (upper panel) or 50 µm (lower panel).

**Supplementary Figure** **9. E-cadherin is upregulated after FAM134B was knockdown in the IHC of xenograft tumors.** Representative IHC images of E-cadherin expression in xenograft tumors. Scale bar, 500 µm (upper panel), 200 µm (middle panel), or 50 µm (lower panel).
